# Supramolecular glasses with color-tunable circularly polarized afterglow through evaporation-induced self-assembly of chiral metal–organic complexes

**DOI:** 10.1038/s41467-023-37331-0

**Published:** 2023-03-24

**Authors:** Fei Nie, Ke-Zhi Wang, Dongpeng Yan

**Affiliations:** Beijing Key Laboratory of Energy Conversion and Storage Materials, College of Chemistry, Key Laboratory of Radiopharmaceuticals, Ministry of Education, Beijing Normal University, Beijing, 100875 P. R. China

**Keywords:** Optical materials and structures, Chemistry

## Abstract

The fabrication of chiral molecules into macroscopic systems has many valuable applications, especially in the fields of optical displays, data encryption, information storage, and so on. Here, we design and prepare a serious of supramolecular glasses (SGs) based on Zn-L-Histidine complexes, via an evaporation-induced self-assembly (EISA) strategy. Metal-ligand interactions between the zinc(II) ion and chiral L-Histidine endow the SGs with interesting circularly polarized afterglow (CPA). Multicolored CPA emissions from blue to red with dissymmetry factor as high as 9.5 × 10^−3^ and excited-state lifetime up to 356.7 ms are achieved under ambient conditions. Therefore, this work not only communicates the bulk SGs with wide-tunable afterglow and large circular polarization, but also provides an EISA method for the macroscopic self-assembly of chiral metal–organic hybrids toward photonic applications.

## Abstract (web summary)

Material designs with multicolor circularly polarized emissions are desirable for photonic applications. Here, the authors report supramolecular glasses based on self-assembled chiral metal–organic complexes with color-tunable circularly polarized afterglow.

## Introduction

Nature has created a large number of bio-organics that possess chiral structures and morphologies, such as amino acids, proteins, enzymes, sugars, DNA and RNA. As indicated by these examples, the bio-organics range in size among molecular, macromolecular, and supramolecular levels^1,2^. Uncovering the self-assembly process of chiral molecules would not only allow an understanding regarding the origin of life, but also provide new opportunities for advances in optical and electronic properties, such as circularly polarized luminescence (CPL). These would allow for extended applications into a variety of areas, including information security, optoelectronic devices, and biological probes, etc^3,4^. Current activities in molecular chirality are mainly limited to the single crystals, gels, and sometimes powders^5–7^; however, the above forms (e.g., individual crystals and powders) are not suitable for the construction of large-scale devices that are needed for practical, real-world applications. Moreover, the random distribution of molecular orientation at the macroscopic level could largely decrease the anisotropy, and thus weaken any circular polarization. For example, in our previous work, we discovered the microcrystals of lanthanide metal–organic frameworks (MOFs) had a low luminescence dissymmetry factor (*g*_lum_, –2.56 × 10^−4^)^8^.

Glassy compounds, such as inorganics^9^, organics^10^, and current MOFs^11^, are a large material family that has made enormous scientific and engineering contributions to human society. These include applications in optics, electronics, heat-insulation, anti-corrosion, and many others^12–15^. We considered that if the chiral molecules could be directly assembled into macroscopic glasses, the integration of a chiral dimension would be more favorable for multi-functional opto-electronics and high-level information storage. However, the fabrication of chiral glasses has been rarely reported. Over the last few years, supramolecular glasses (SGs) have drawn widespread attention, due to their advantages of combining the beneficial aspects of low-molecule-weight building blocks, good film-forming ability, high thermal stability, and easy processability^16–19^. The monomeric species based on π-electron systems typically need to be designed in nonplanar or irregular shape, which may inherently resist easy molecular packing and thus ready crystallization, and particularly the molecular geometries (including star, branch, tetrahedron, spiro, and ring) are preferred^18,19^. Up to now, the primary formation of SGs has relied on conventional steps including melting-quenching technique and physical vapor deposition^20–23^. There exist some intrinsic drawbacks to these preparation methods, including the possible quenching of photo-activities (e.g., luminescence) at high temperature, the poor transparency ascribed to the decomposition of organic linkers during processing, as well as the difficulty in the precise control of shapes. Additionally, there are limited categories of building blocks in the as-reported SGs, such as ureidopyrimidinone derivatives^24,25^, triazine derivatives^26,27^ and azobenzenes derivatives^28,29^. Collectively, these deficiencies have made the fabrication of high-performance SGs difficult and thus restricted the embedding of SGs into various applications. Therefore, developing a new synthetic approach and alternative monomeric species for use in transparent photoactive SGs with both large size and various dimensionalities remains highly desirable.

Recently, molecular persistent afterglow—which is beyond common fluorescence—has mainly included thermally activated delayed fluorescence and room temperature phosphorescence (RTP). Due to the obvious applications, it has generated broad attention in the fields of sensors, anti-counterfeiting, displays and bioimaging^30–32^. With the integration of molecular charity and afterglow, the limited examples of circularly polarized afterglow (CPA) have been demonstrated in systems including co-crystal, host–guest structure, and chiral chain engineering^33–35^. Nevertheless, an efficient and universal approach to achieve simultaneous high circular polarization, ultra-long afterglow lifetime, and color-tunable CPA does not exist. Also, it remains challenging to obtain CPL-active thin film materials with long-lived afterglow toward photonic applications.

**Fig. 1:**
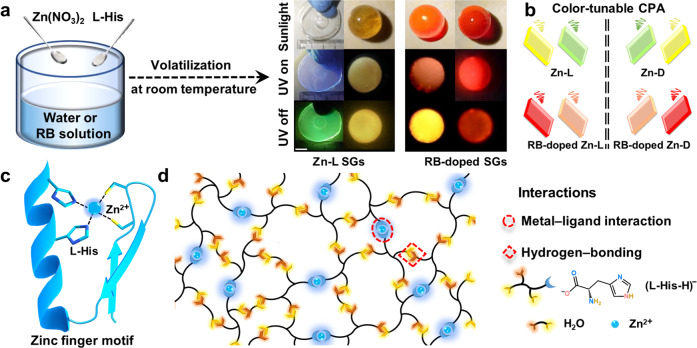
Schematic illustration of Zn–L SGs fabrication. **a** Preparation of Zn–L and RB-doped SGs (RB: Rhodamine B), and photos of the obtained Zn–L and RB-doped SGs in the shape of a sphere and/or a round sheet taken under the sunlight, and before and after UV-light turned off. Scale bar: 0.8 cm. **b** Multicolored CPA emissions from chiral SGs. Spiral lines represent CPA-active signals. **c** C2H2-type zinc finger motif. **d** Schematic representation of the SGs assembly.

L-Histidine (L-His), as an essential amino acid in human body, has natural chirality and photoactivity. It is also rich in supramolecular sites that are involved in self-assembly, driven by reversible and dynamic metal-ligand interaction and hydrogen-bonding^36–38^. Notably, the organic-metal binding between L-His and zinc(II) ion plays a vital role in the zinc finger proteins, where the classic motif has the zinc(II) ion coordinates to two L-His residues and two cysteine residues^39–42^. Besides, through the interaction with L-His residues, zinc(II) ion can initiate intrinsically disordered protein (IDP) oligomerization. This may lead to the formation of insoluble disease-related aggregates, such as the aggregation of L-His-rich amyloid-β-peptides (Aβ), which is a major pathological event in Alzheimer’s disease^43–46^. Inspired by the structure and interactions found in zinc finger proteins, herein, we have designed and fabricated a series of Zn-L-Histidine (Zn–L) SGs with multicolored photoemissions through a facile and environment-friendly self-assembly strategy in solution (Fig. 1). Although extensive experimental and theoretical efforts have been devoted to the biomimetic process of Zn–L complexes in both crystal and hydrogel^39–43^, the SGs based on Zn–L metal–organic complexes have yet to be reported.

Interestingly, the triple-mode (including solute content, temperature, and excitation wavelength) dependent ultralong RTP emissions were observable, which benefitted from the nitrogen and oxygen atoms contributing to rich energy levels for the tunable afterglow properties^47^. Moreover, unusual orange–red and red CPA emissions could be achieved by doping Rhodamine B (RB) into the Zn–L SG matrix, as a result of efficient phosphorescence resonance energy transfer (PRET). By virtue of the multicolored CPA emissions, high transparency, and easy film-forming ability of these SGs, their potential applications in optical displays were further demonstrated. These findings not only provide the foundation for the chiral molecular glasses with color-tunable CPA, but also supply a potentially universal, bottom-up approach for optical SGs.

## Results

### Preparation of metal–organic SGs

Based on the model of the zinc finger motif, Zn(NO_3_)_2_·6H_2_O and L-His with a molar ratio of 1:2 were firstly dissolved in deionized water. The solution then slowly evaporated at room temperature for several days, causing the formation of a very viscous Zn–L hydrogel (Supplementary Fig. 1). Prolonged drying of the hydrogel resulted in the volatilization of excessive solvent. Strikingly, this prolonged drying at room temperature finally led to the formation of Zn–L SGs with super smooth and flat surface and high stiffness (Supplementary Fig. 2). Comparatively, the preparation at high temperature (i.e., 90 °C) led to the formation of the glassy sample with numerable honeycombed passages as well as the green persistent luminescence property (Supplementary Fig. 3). Promisingly, the SGs in round-sheet and spherical shapes with multi-colored emissions were fabricated at large scales by casting the hydrogels into silicon and plastic molds, respectively (Supplementary Movie 1–4). There is sound evidence that the SGs are harmless to humans, since the Zn–L hydrogel has many beneficial properties for human skin^48^. Alternatively, the SGs could also be prepared by slow evaporation of a mixture of water and a volatile organic solvent (e.g., methanol, ethanol, acetone, and methyl cyanide, volume ratio: 3:1). In contrast, the bulk Zn–L single crystals were obtained from a mixture of water and involatile pyridine (volume ratio: 3:1). This may be because that as the volatilization proceeded, a considerably smaller amount of volatile organic solvent was trapped in the SG matrix and formed strong hydrogen bonds with the Zn–L complexes. Comparatively, large quantities of pyridine molecules mainly formed weak van der Waals forces with the complexes. These results indicated that solvent-bridged hydrogen bonding greatly promoted the formation of a polymeric SG network. Additionally, we speculated that the water not only served as a good solvent but also acted as a perturbation agent to prevent crystallization.

When we only added L-His or replaced L-His with other amino acids (e.g., L-Alanine, L-Phenylalanine, L-Tryptophan, or L-Cysteine), only powders or crystals were obtained in water phase. This result confirmed the important role of metal-ligand interactions in regulating the SG formation. Moreover, the lack of an imidazole group for other amino acids was responsible for their weak coordinating ability with Zn^2+^. Consequently, the synergistic effect of the multiple interactions—especially hydrogen bonding and metal-ligand interactions among L-His, Zn^2+^ and H_2_O—significantly drove the formation of the SG network structure.

### Structural characterizations

**Fig. 2:**
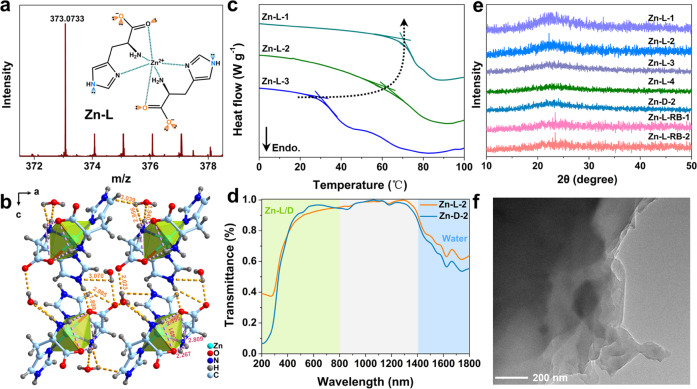
The molecular structure of the Zn–L complex, and the thermal properties and morphologies of the SGs. **a** The HR-ESI-MS spectrum of the Zn–L complex. The inset was the illustration of the molecular structure of the Zn–L complex. **b** The hydrogen-bonding network in the Zn–L crystal structure. The orange and purple dashed lines respectively represent intra/intermolecular hydrogen bonds. There were intra/intermolecular hydrogen bonds between the complexes, as well as hydrogen bonds between the complexes and the solvent water molecules. The Zn–L complex showed a twist configuration in the crystal, owing to the presence of alkyl chains. **c** The DSC traces of Zn–L-1/2/3 SGs. **d** UV-Vis-NIR transmittance spectra results for Zn–L/D-2 SGs. The Zn–L/D-2 SGs were highly transparent (above ~90%) in the visible and NIR ranges (gray area). Their bands in the cyan and blue areas originated from the Zn–L complexes and water absorption, respectively. **e** The PXRD patterns of Zn–L-1/2/3/4, Zn-D-2 and Zn-L-RB-1/2 SGs. Note: Zn–L-4 glass corresponds to Zn–L mass content of 90.5 wt%. **f** The representative TEM image of Zn–L SG.

The structures of the target SGs were next examined using nuclear magnetic resonance (NMR), high-resolution electrospray ionization mass spectrometry (HR-ESI-MS), X-ray photoelectron spectroscopy (XPS) measurements, element analysis, and Fourier-transformed infrared (FT-IR) spectroscopy. The ^1^H NMR spectrum for Zn–L glass illustrated that when compared with those of pristine L-His, the protons located at the imidazole rings were notably shifted downfield (Supplementary Fig. 4), which provided support for the strong coordination of imidazole with Zn^2+^ ^39^. Both the amino and carboxy groups were also possibly involved in the coordination, as the protons located at the carbon atoms were deshielded by a relatively smaller extent. The HR-ESI-MS spectrum for Zn–L glass revealed a peak with m/z = 373.0733, which was in full agreement with a 1:2 Zn-coordinated structure and provided strong evidence for the high purity of the produced SGs (Fig. 2a). Also, the high purity of the SGs could be further supported by the element analysis revealing 27.45% carbon, 21.18% nitrogen and 3.308% hydrogen, as well as almost identical photophysical properties of SGs prepared from L-His powders purchased from different manufacturers (Supplementary Fig. 5).

The N 1*s*-XPS spectrum of Zn–L glass showed a peak at binding energy of 399.5 eV (Supplementary Fig. 6), attributing to the presence of Zn-N coordination in the SG network^49^. Moreover, as shown by the FT-IR spectrum of Zn–L SG (Supplementary Fig. 7a), the appearance of two new Zn-N peaks at 530 and 623 cm^–1^, together with the shift of the symmetric C = O stretching (at 1506 cm^–1^) of the carboxylate group relative to that (at 1587 cm^–1^) of pristine L-His, accounted for the coordination of the amino, imidazole, and carboxylate groups with Zn^2+^, respectively^41^. The shifts and broader bands of N–H stretching (at 3128 and 3425 cm^–1^) of the amino groups in Zn–L SGs, relative to that (3470 cm^–1^) of L-His powder, may have resulted from the involvement of amino groups with stronger hydrogen bonding. The very close ^1^H NMR, XPS and FT-IR spectra between the SG and the single crystal form confirmed their highly similar chemical environment and intermolecular interactions (Fig. 2b, and Supplementary Fig. 4, 6–8 and Table 1). Based on these results, we proposed an octahedral structure for the Zn–L complex in the SG. More specifically, the imidazole, carboxylate and amino groups of two L-His ligands were involved in the coordination with Zn^2+^ (as shown in the inset of Fig. 2a, Supplementary Fig. 9).

Differential scanning calorimetry (DSC) and thermogravimetric analysis (TGA) were used to determine the thermal properties of the SGs. For samples with different Zn–L mass contents (Zn–L-1 (98.8 wt%), Zn–L-2 (97.0 wt%), and Zn–L-3 (94.7 wt%)), it was revealed that their glass transition temperature (*T*_g_) values were 70.3, 62.0 and 29.7 °C, respectively, suggesting the solvent content tailored the *T*_g_ of the SG (Fig. 2c). This could be understood that the higher *T*_g_ in the DSC curve of the supramolecular system was associated with stronger non-covalent interactions and corresponded to a more stable cross-linking network structure^19^. Upon doping with RB dye, the *T*_g_ exhibited a corresponding decrease (Supplementary Fig. 10a), possibly due to that the coordination between trace RB and Zn^2+^ caused the disturbance of the short-range ordered structure of the SG^50^. Additionally, there was a decrease of *T*_g_ by increasing or decreasing of the metal/L-His ratios (Supplementary Fig. 10b), perhaps because the uncoordinated metal ions and L-His molecules as the impurities, deteriorated network connectivity in the SG matrix. This illustrated that the considerably higher SG structure stability was achieved when the molar ratio of Zn(NO_3_)_2_ to L-His was 1:2. It was noteworthy that the change of the metal salt Zn(NO_3_)_2_·6H_2_O into ZnCl_2_, Zn(ClO_4_)_2_, ZnC_2_O_4_, Cd(NO_3_)_2_, or even Eu(NO_3_)_3_ and Tb(NO)_3_ also led to the formation of stable SGs (Supplementary Fig. 10c, d). No *T*_g_ signal was reflected in the DSC curve of the pristine L-His (Supplementary Fig. 10b), indicating the Zn^2+^-binding with L-His was the indispensable factor in SG formation.

In the TGA traces of Zn–L-1/2 SGs (Supplementary Fig. 11a), the weight loss of less than 5% before 250 °C was mainly related with the gradual loss of hydrogen-bonded water molecules, which indicated that the enhanced non-bonded interactions were favorable to the construction of a highly stable polymeric structure. Therefore, the self-assembled systems basically existing in the form of glasses above 200 °C, manifested that this wet-chemistry method effectively suppressed the tendency towards crystallization observed in many reported SGs that were synthesized by traditional methods at high temperature between the melting point and *T*_g_^19^. Impressively, Zn–L-3 SG exhibited less weight loss above 130 °C than Zn–L crystal, despite their similar water contents (Supplementary Fig. 11c). Compared with the limited intra/intermolecular interactions in the crystal (Supplementary Data 1), higher synergistic non-bonded interactions in the SG could effectively drive the assembly of the molecular units toward a higher thermodynamic stability. The identical thermal stabilities of as-synthesized Zn–L/D-2 glasses demonstrated that the variation of molecular configuration had almost no effect on SG stability (Supplementary Fig. 10e, 11d). Taken together, the TGA results coincided with the DSC data, and both supported the presence of strong non-covalent interactions. This was especially true for metal-ligand and hydrogen bonding interactions, which collectively improved the polymeric connection and thermal stability of the SGs. Ultimately, this improvement would allow for their applications across a wide temperature range.

The microstructures of these transparent samples were next investigated by powder X-ray diffraction (PXRD), transmission electron microscopy (TEM), and optical microscopy (Fig. 2d, and Supplementary Fig. 12). The small and broad peaks in the PXRD patterns implied the amorphous state and long-range disordered structure of the as-prepared SGs (Fig. 2e, and Supplementary Fig. 13). The TEM imaging coupled with the elemental mapping of the Zn–L glass suggested enrichment of the thin 2D layers by Zn, C, N and O (Fig. 2f, and Supplementary Fig. 14). We deduced that the existence of the layered structure was possibly due to the assembly of dispersed L-His and Zn^2+^, driven by multiple non-covalent interactions. This occurred at the air/liquid/solid interface upon further solvent evaporation, and the layer-to-layer interactions were largely maintained by hydrogen-bonding between the inserted water and the complexes. Furthermore, the bubbles trapped in the Zn–L SGs and observed under the optical microscopy were mainly caused by the evaporation of solvent molecules, and they gradually disappeared as the volatilization proceeded (Supplementary Fig. 15). Given these, the whole process from the coordination between ligands (L-His) and metal ion (Zn^2+^) to the final cross-linked network could be viewed as an evaporation-induced interfacial self-assembly process.

### Color-tunable persistent luminescence

**Fig. 3:**
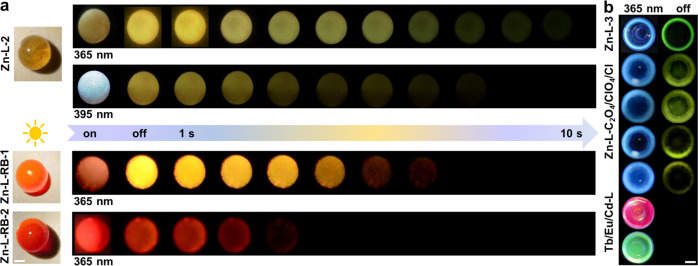
Representative images of ultralong RTP observed in the glasses. **a** Images of spherical Zn–L-2 and Zn-L-RB-1/2 SGs taken before and after the excitation turned off across a time scale of 0 to 10 s. **b** Images of thin Zn–L-3, Zn-L-Cl/ClO_4_/C_2_O_4_ and Cd/Eu/Tb-L SGs taken before and after 365 nm irradiation turned off under ambient conditions. Scale bar: 0.8 cm.

Interestingly, for the macroscopically globular Zn–L-2 SG, green-yellow emission was observed after ceasing irradiation using a 365 nm ultraviolet (UV) lamp, and faded gradually with notably persistent luminescence, lasting approximately 10 s under ambient conditions (Fig. 3a, and Supplementary Movie 2). When the excitation source was switched to 395 nm, persistent yellow luminescence was captured. The emission color varied with the excitation wavelength also appeared in the round-shape Zn–L-2 glass sheet (Supplementary Movie 1). Besides, blue and green emissions from thin Zn–L-3 glass could be respectively observed with the naked eye before and after ceasing excitation at 365 nm (Fig. 3b). Impressively, once the RB dye was doped into the SGs, persistent orange–red and red emissions in the time range of 0–7 s were observable from the resulting bulk globular Zn-L-RB-1/2 glasses, respectively (Supplementary Movie 3, 4). Moreover, thin Zn-L-Cl/ClO_4_/C_2_O_4_ and Cd-L glasses all exhibited blue and green-yellow emissions before and after ceasing the 365 nm excitation, respectively. Eu/Tb-L glasses glowed red and green respectively when illuminated by a 365 nm lamp (Fig. 3b). Therefore, by doping dye or using different Zn/Cd and rare-earth ions as precursors to prepare SGs, multi-colored observable luminescence could be achieved across a wide emission range from blue to red.

To understand the origin of the observable afterglow of SGs, we first studied the photoluminescence (PL) properties of the pristine L-His. The decay curve of L-His powder showed the photoemission with a relatively short lifetime of 22.3 µs at room temperature (Supplementary Fig. 16a, b). The phosphorescence feature of L-His powder could also be confirmed by the decrease of the delayed emission intensity with the increased temperature (Supplementary Fig. 16c). The short phosphorescence emission of L-His powder was in stark contrast to the bright afterglow of Zn–L SGs at room temperature. It was deduced that the rotation and vibration of L-His molecules in the glass matrix could be greatly restricted by multiple noncovalent forces (including metal-organic coordination and hydrogen-bonding interactions), which caused the suppression of non-radiative transition of the triplet excitons. Additionally, the heavy atom effect of Zn^2+^ ion could enhance the spin–orbit coupling (SOC) to facilitate intersystem crossing (ISC), and hence yielded ultralong-lived RTP of Zn–L SGs.

**Fig. 4:**
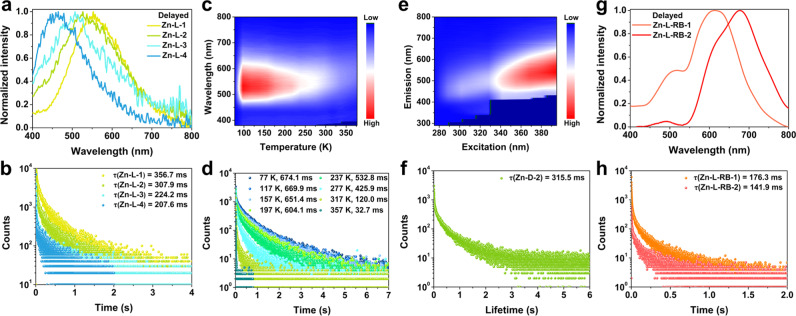
Photophysical properties of Zn–L SGs and RB doped SGs. **a**, **b** Delayed PL spectra and lifetime profiles of Zn–L-1/2/3/4 SGs excited by 365 nm at room temperature. **c**, **d** Delayed PL mapping spectra and lifetime profiles of Zn–L-2 SG excited by 365 nm at different temperatures. **e** Delayed PL spectra of Zn–L-2 SG excited by different wavelengths at room temperature. **f** Phosphorescence lifetime decay profile of Zn-D-2 SG excited by 365 nm at room temperature. **g**, **h** Delayed PL spectra and lifetime profiles of Zn-L-RB-1/2 SGs excited by 365 nm at room temperature.

To further study the photophysical properties, we next systemically characterized the prompt and delayed PL spectra of the SGs. It was observed that the Zn–L SGs exhibited solute content-, temperature-, and excitation-dependent emissions (Supplementary Fig. 17). In their prompt PL spectra, these transparent SGs displayed dominant emissive peaks from 430 to 500 nm with lifetimes at the nanosecond level (Supplementary Fig. 18). Additionally, the emission in the long-wavelength range of each prompt PL spectrum (e.g., from 510 to 800 nm for Zn–L-2 glass) was mainly attributed to the ultralong RTP. This was in accordance to the descriptions regarding many reported molecular afterglow materials^30–35^. Prominently, from Zn–L-4 to Zn–L-1 SGs, they exhibited a solute content-dependent afterglow, showing typical emission bands ranging from 465 to 555 nm with color variations from blue to yellow, and ultralong lifetimes from 207.6 to 356.7 ms (Fig. 4a, b, and Supplementary Table 2). In the case of high Zn–L complexes content, we deduced that the enhanced non-bonded interactions contributed to reduce the band gap. Also, the enhanced interactions greatly promoted the ISC process, facilitating the production of the triplet excitons.

Strikingly, temperature- and excitation-dependent delayed emissions were achieved in the prepared SGs. Considering Zn–L-2 glass as an example, the emission maximum shifted from 529 to 579 nm as the temperature changed from 77 to 357 K, accompanied with the afterglow color changing from green-yellow to orange (Fig. 4c, and Supplementary Fig. 19b, c). The decay curves present in Fig. 4d revealed the lifetime up to 674.1 ms at 77 K. The temperature-dependent red shift was attributed to that the increase of temperature could reduce the population of the twisted configuration of the Zn–L complex. In addition, it may also have simultaneously increased the thermal relaxation of the twisted form to the planar form, which contributed to the narrowing of energy gap^51^. Decreases in emission intensity and lifetime with increased temperature could be ascribed to the disruption of non-covalent interactions at high temperatures, and the gradual release of inhibition of the molecular rotation within the glass matrix^52^, which also provided solid evidence for the RTP characteristic of the SGs^30^. Moreover, the more significant red shift observed in the delayed PL spectrum across 297–377 K compared with that of 77–297 K was related to the volatilization of free water molecules in the glass (Supplementary Fig. 11a). Similar phenomenon also appeared in the prompt PL spectrum (Supplementary Fig. 19a, c). These findings were in agreement with the red-shifted emissions of the SGs that had increased Zn–L contents. Furthermore, with varying the excitation wavelength from 275 to 395 nm, we observed gradually redshifted RTP with emission bands from 460 to 545 nm coupled with increased intensities (Fig. 4e, and Supplementary Fig. 20a, b). Taken together, the Zn–L-2 SG possessed promising wavelength-dependent phosphorescence (WDP) characteristic. We deduced that multiple excited states were responsible for this color-tunable ultralong phosphorescence (Supplementary Fig. 20c), which could be further verified by the following theoretical calculation. Expectantly, the emission performance of Zn-D-2 glass was similar to that of Zn–L-2 glass (Fig. 4f, and Supplementary Fig. 21 and Table 2), indicating that the change of molecular configuration was of weak effect on the optical properties of the SGs.

Notably, long-wavelength emissions were obtained from RB-doped SGs, owing to the efficient energy transfer from Zn–L complexes to RB via PRET process. The prompt PL spectra of Zn-L-RB-1/2 SGs exhibited approximately two emission bands ranging from 320–537 nm and 537–800 nm (Supplementary Fig. 22), which originated from emissions of the Zn–L complexes and RB in the SGs, respectively. In the delayed mode, the Zn-L-RB-1 SG exhibited one small shoulder band in the range of 400–537 nm and one band with a peak at 615 nm (Fig. 4g). Similarly, for Zn-L-RB-2 SG, there was one weak band ranging 416–537 nm and the other red-shift band at 678 nm. Impressively, the decay curves of the Zn-L-RB-1/2 glasses showed ultralong afterglow lifetimes of 176.3 ms (at 615 nm) and 141.9 ms (at 678 nm), respectively (Fig. 4f, and Supplementary Table 2). Additionally, persistent orange–red and red emissions could be clearly observed from Alizarin Red S (ARS) and Erythrosin B (EB) doped glassy samples, respectively (Supplementary Fig. 23). These results indicated that the broad phosphorescence emission of Zn–L complexes in the glassy samples allowed a wide selection of energy accepters for color-tunable afterglow emissions.

The emission behaviors of Zn-L-Cl/ClO_4_/C_2_O_4_ glasses were similar to those of Zn–L-2 glass (Supplementary Fig. 24), indicating that the anions trapped in the polymeric network had negligible influence on the optical properties of the SGs. In addition, Cd-L glass displayed both prompt and delayed emission bands similar to those of the Zn–L-2 glass, but with a shorter afterglow lifetime (114.1 ms) than the latter (307.9 ms) (Supplementary Fig. 25a, b). This was possibly due to the enhanced heavy atom effect of Cd^2+^ compared with that of Zn^2+^ ^53^. Moreover, Eu-L and Tb-L glasses showed red and green phosphorescent emissions with decay lifetime values of 445.8 µs and 1.2 ms, respectively (Supplementary Fig. 25c, d). This was perhaps because the L-His acting as a ligand in the supramolecular systems became a promising host and served as “antennas” to achieve high-efficiency sensitization for the lanthanide ions^54^.

In striking contrast, the crystalline Zn–L sample exhibited almost invariable phosphorescent emissions at approximately 510 nm under the irradiation at different excitation wavelengths (Supplementary Fig. 26a, b). This was likely due to the considerably homogeneous microenvironment in the crystal. Compared to the heterogeneous microenvironment in the SGs, this homogenous system was not conducive to the formation of species with multiple excited states. Besides, the phosphorescence decay lifetime of the Zn–L crystal was rather short to be 6.1 ms (Supplementary Fig. 26c). These findings clearly demonstrated that the high stiffness of the microstructure played a crucial role in the long-afterglow emissions of the SGs (Supplementary Fig. 2)^55^.

### Color-tunable CPA properties

**Fig. 5:**
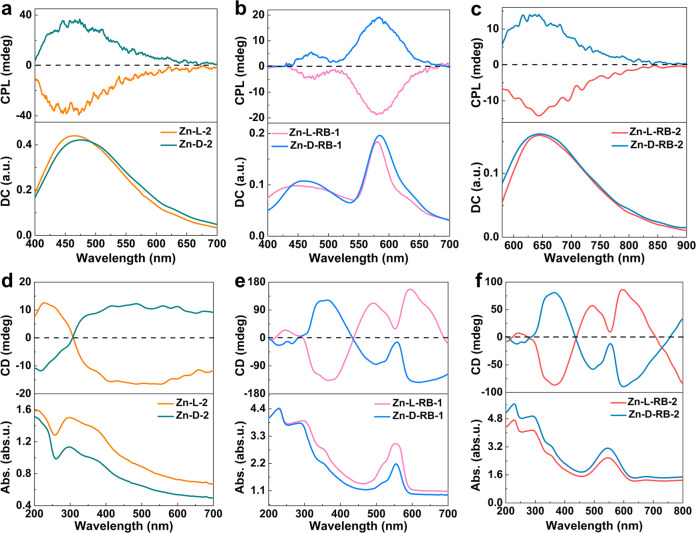
The CPL properties of the SGs. **a**–**c** The CPL spectra of Zn–L/D-2, Zn–L/D-RB-1 and Zn–L/D-RB-2 SGs excited by 365, 380, and 400 nm, respectively. “DC” is analogous to “PL intensity”. **d**–**f** The CD and UV-Vis absorption spectra of these SGs.

The CPL spectra revealing the chirality of excited states of the SGs were further examined. In particular, the strong CPL emissions of Zn–L-2, Zn-L-RB-1/2 and their corresponding enantiomers-formed SGs as typical examples intrinsically coincided with their corresponding prompt PL spectra (Fig. 5a–c). Thus, the CPL emissions were assigned in terms of their prompt and delayed PL spectra. Given this, it was reasonable to conclude that color-tunable CPA emissions were successfully realized in these SGs. Clearly, the enantiopure forms of SGs presented excellent mirror-image CPL signals. Moreover, the CPA wavelength was red shifted with the emission color changing from green–yellow across orange–red to red. This was achieved by increasing the RB content from Zn–L/D-2, Zn–L/D-RB-1, to Zn–L/D-RB-2 SGs. The CPL magnitude was evaluated by the optical dissymmetry factor, which can be obtained from *g*_lum_ = 2(I_L_-I_R_)/(I_L_ + I_R_), where I_L_ and I_R_ are the emission intensities of left and right CPL, respectively^35^. These SGs exhibited prominent circularly polarized fluorescence and phosphorescence emissions with a maximum dissymmetric factor of 9.5 × 10^–3^ (Supplementary Fig. 27), which was either higher or comparable to those for state-of-the-art CPA-active materials^33–35^.

Moreover, the chirality of the enantiomeric SGs in the ground states was also investigated by using circular dichroism (CD) spectra. As depicted in Fig. 5d–f, the CD spectra of Zn–L/D-2 and RB-doped SGs presented a good mirror-image relationship with alternating positive and negative Cotton effects from 200 to 700 nm^33–35^. Particularly, compared with Zn–L/D-2 SGs, the doped glasses showed additional absorption bands from 450 to 580 nm arising from the feature absorption of RB (Supplementary Fig. 35a). In contrast, the L/D-His powders showed very weak CD signals and negligible CPL emissions (Supplementary Fig. 28). The maximum |*g*_lum_| values realized in the glassy samples were notably higher than that (ca. 1 × 10^–4^) of the Zn–L single crystal (Supplementary Fig. 29). These results corroborated the importance of the SGs’ macroscopic self-assembly for amplifying the circular polarization effect.

### Mechanism for ultralong RTP and energy transfer

For the Zn–L complexes in aqueous solutions (1 × 10^−2^ and 1 × 10^−3^ M) at 77 K, there were nearly invariable emissions when excited at different wavelengths, and their decay curves gave phosphorescence lifetimes of 5.92 and 3.87 µs, respectively (Supplementary Fig. 30a–c). Strikingly, upon increasing the concentration to 1 × 10^−1^ M, the aqueous solution of Zn–L complexes at 77 K exhibited an obvious WDP property, and the decay curve gave an ultralong afterglow lifetime up to 632.8 ms (Supplementary Fig. 30d, e). These findings were in agreement with the WDP and afterglow properties of the solid SGs at room temperature (Fig. 4a, b). Thus, we primarily deduced that the boosted persistent luminescence for the frozen solution of Zn–L complexes was ascribed to the aggregation-induced phosphorescence enhancement (AIPE) mechanism. More specifically, the aggregation based on multiple intra/intermolecular interactions not only narrowed the band gap, but also led to the formation of different clusters with plentiful triplet energy levels. This resulted in the increased ISC rate, and ultimately the enhanced persistent phosphorescence lifetime^56^. However, for the pristine L-His molecules in aqueous solution (1 × 10^−1^ M) at 77 K, there was no WDP behavior, and its decay curve showed a short phosphorescence lifetime of 11.2 µs (Supplementary Fig. 31), which was closer to RTP lifetime of the solid L-His (Supplementary Fig. 16a, b).

**Fig. 6:**
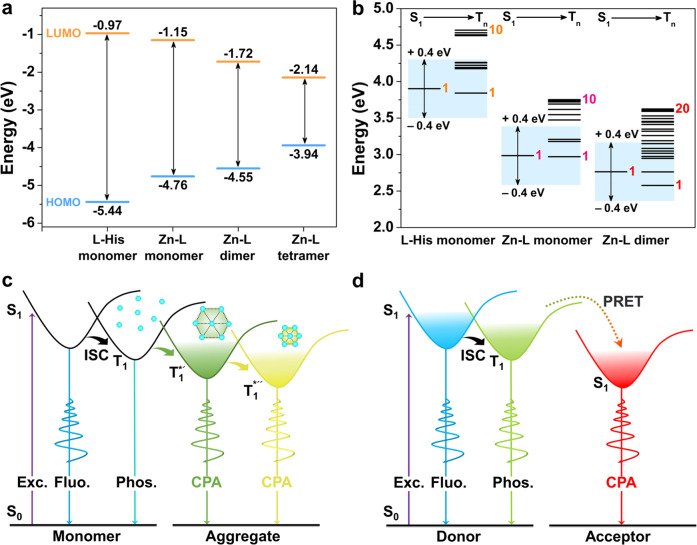
Theoretical analyses of color-tunable CPA from SGs. **a** Calculated energy bandgaps for the selected L-His monomer extracted from the L-His single crystal (CCDC: 1206542), and Zn–L monomer and aggregates extracted from Zn–L single crystal. The blue and orange lines illustrate the energy values of HOMO and LUMO, respectively. **b** TD-DFT calculated energy level diagrams of selected L-His monomer, as well as Zn–L monomer and dimer in L-His and Zn–L single crystal, respectively. **c**, **d** Proposed mechanisms of the CPA emissions from Zn–L SGs and RB doped SGs. Cyan ball represents the Zn–L complex. The black dotted line symbolizes non-covalent interaction between the complexes—the shorter the line, the stronger the interaction. S_0_, S_1_, and T_1_ represent the ground state, the lowest singlet, and triplet excited states, respectively. T_1_*' and T_1_*'' are the stabilized triplet excited states originating from varied emission species with different aggregation degrees, respectively. Fluo. and Phos. are the abbreviations of fluorescence and phosphorescence, respectively. ISC and PRET are the abbreviations of intersystem crossing and phosphorescence resonance energy transfer, respectively. The color of the spiral lines and the lines representing the energy states is similar to the corresponding luminous color.

These results highlighted the indispensability of the metal-ligand coordination for the WDP behaviors and long-afterglow properties of the frozen solution of Zn–L complexes and the SGs. This was because the restricted rotation/vibration of L-His molecules as luminophores reduced the non-radiative transition. Moreover, the red-shifts of both prompt and delayed emissions occurred with increasing concentration of Zn–L complexes in frozen solutions (Supplementary Fig. 32). This was consistent with the bathochromic emissions in the SGs with increasing solute content (Fig. 4a, and Supplementary Fig. 18). We inferred that the red shifts in the frozen solutions and solid-state glasses were mainly due to the narrowing of the energy bandgap with the increase of aggregation degree (Fig. 6a).

To support the proposed mechanism responsible for the afterglow emissions of the SGs, a series of spectra were next monitored at room temperature. It was observed that for Zn–L complexes and L-His aqueous solutions, the prompt and delayed emissions increased with the increasing concentrations from 1 × 10^−5^ to 1 × 10^−1^ M, respectively (Supplementary Fig. 33a–d). In this condition, gradually increased absorption of Zn–L complexes aqueous solution occurred in a larger range (250–450 nm) than that of L-His (250–320 nm) (Supplementary Fig. 33e, f). This implied that the formation of the complexes was more favorable for light absorption. Moreover, we speculated that the intense absorption bands from 250 to 320 nm detected for Zn–L complexes solutions were assigned to the spin-allowed ligand-centered (LC) transition of L-His fragments. The absorption bands have stemmed from the transition from highest occupied molecular orbital (HOMO) to the lowest unoccupied molecular orbital (LUMO). The calculated HOMO-LUMO transitions of the Zn–L monomer and dimer fell right into this range (Fig. 6a).

In addition, enhanced absorption (250−300 nm) and emission were also observed for both Zn–L complexes and L-His solutions (1 × 10^−5^ M) of water (as a good solvent) and methyl cyanide (MeCN) (as a poor solvent) with the increase of MeCN fraction (*f*_w_) (Supplementary Fig. 34). In particular, the prompt emission maxima in the range of 275−300 nm for the mixed solution of Zn–L complexes presented a bathochromic shift with increasing *f*_w_. Moreover, a new strong band appeared when *f*_w_ increased to 90%. This may be because as the increase of *f*_w_, the aggregation degree of the complex enhanced, with the gradual emergence of its dimers, trimers, tetramers, and so on. Collectively, their occurrence sequentially reduced the energy gap and resulted in the red shift. The aggregation state also led to the appearance of a new peak with lower energy. It was worth noting that the increased degrees of absorption and emission intensities of the Zn–L complexes in both aqueous and mixed solutions were greater than those of L-His. This was probably ascribed to the presence of coordination interactions, which further restricted the rotation of L-His molecules and led to stronger AIPE. Impressively, upon coordinating with Zn^2+^, the changes of absorption and emission spectra for L-His solution may provide a deeper understanding of the dynamical aggregation of L-His-rich proteins (e.g., IDP), as well as the regulation of corresponding biological processes through metal ion^2+^-binding L-His^43–46^.

We next investigated the energy transfer properties of RB-doped SGs (Supplementary Fig. 35). For the delayed PL spectra of the SGs doped with different RB contents, as the molar ratio of RB to Zn–L complexes increased from 0 to 6 × 10^–8^, the green phosphorescence intensity decreased, while the luminescence ranging from 553 to 800 nm increased distinctly (Supplementary Fig. 35b). Meanwhile, the afterglow peak ascribed to RB shifted from 540 nm to 680 nm. Thus, the Zn–L complexes along with RB comprised a potentially efficient PRET pair, in which RB absorbed the phosphorescence energy from the Zn–L complexes and then emitted red-shift afterglow through PRET^57–61^. The PRET efficiency between the Zn–L complexes and RB could be calculated according to the equation: E = 1 − I/I_0_, where I and I_0_ are the intensities of the phosphorescence peaks of the donor Zn–L complex with and without the acceptor RB, respectively. The corresponding PRET efficiencies reached 66.6% and 95.7% for the Zn-L-RB-1/2 SGs, respectively. The achieved efficiency value (95.7%) was higher than those of many previously reported PRET systems^57–61^. This may be benefitted from the large spectral-overlapping integral between the phosphorescence emission of the Zn–L complexes and the absorption of RB (Supplementary Fig. 35a)^57^.

### Theoretical calculations

To understand the photophysical processes from an electronic structure view, a set of theoretical investigations including frontier molecular orbitals, time-dependent density functional theory (TD-DFT) calculations and surface electrostatic potential (ESP) analyses on the monomer and/or selected aggregates of the Zn–L complex and L-His were conducted. For the Zn–L complex, the HOMOs and LUMOs in the monomer were mainly localized on the carboxylate and amino groups, and imidazole groups of the two ligand factions, respectively (Supplementary Fig. 36). In the complex dimer and tetramer, the HOMOs and LUMOs were respectively delocalized on these groups belonging to different monomers^33^. This effect was in favor of narrowing the energy bandgap and leading to the formation of different clusters with diverse triplet energy levels^33,62^, corresponding to the WDP properties and the proposed AIPE mechanism. As expectedly, the coordination of L-His to Zn^2+^ and the increase of the complex aggregation size resulted in a narrower HOMO-LUMO energy gap (Fig. 6a), which underscored the occurrence of more available electronic transitions between the ground and excited states. These results coincided with the gradually decreased excited energy levels, owing to the coordination of L-His and the aggregation of Zn–L complexes (Fig. 6b).

To gain deeper insight into the impact of the complexes’ aggregation on the ultralong afterglow of the glasses, the TD-DFT calculations were next conducted (Supplementary Table 3–5). As shown by the simulated excited state energy levels, there were three triplet states presenting singlet and triplet splitting energy (Δ*E*_ST_) less or greater than 0.40 eV for enabling possible transition of excitons from singlet to triplet states in the Zn–L monomer based on the energy gap law (Fig. 6b)^63,64^. Significantly, the total number of plausible ISC channels in the dimer was increased to 10, thereby more efficiently promoting the ISC process. Furthermore, the calculations suggested that the lowest singlet state (S_1_), and triplet states as plausible ISC channels in the Zn–L monomer and dimer were characterized by the multiple energy levels. This was aided by the heteroatom incorporation of nitrogen and oxygen atoms as required by EI-Sayed’s rule^34^, which usually generates large spin SOC values for efficient ISC and ultralong afterglow lifetime.

The ESP analyses for the L-His monomer, Zn–L monomer and Zn–L dimer were calculated to visualize and comprehend the intermolecular interactions (Supplementary Fig. 37). We observed that in the Zn–L complex system, the region around the carboxylate group represented the most negative potential region (blue), and the most positive potential site (red) was largely around the hydrogen atom linked with the nitrogen atom of the imidazole group. This implied the potential formation of strong intermolecular hydrogen bonding between the carboxylate and imidazole groups.

Molecular dynamic simulations were also performed to simulate the intermolecular interactions among the chemical components in supramolecular Zn-L–H_2_O systems with typical Zn–L complexes mass portions (94.7 wt%, 97.0 wt% and 98.8 wt%). The main purpose of the simulations was to examine the solute–solute and the solute–solvent interactions using radial distribution function (RDF), which describes the density variations with the change of distance relative to a selected reference particle. This allows for the evaluation of the chance of finding a particle from the selected atom or molecule at a certain distance in the simulated solution^65^. On the basis of RDF *g*(r), the larger the *g*(r) value is, the greater the appearance possibility of the interactions between different particles would be. Given this, the RDF peaks before 3.5 Å and in the range of 3.5–5.0 Å were related to hydrogen bonding and van der Waals force, respectively. By detecting the distance between oxygen (nitrogen) and hydrogen atoms located at the carboxyl, imidazole, or amino groups of either the complex or water molecule (signed in Supplementary Fig. 38), we explored the hydrogen bonds in the systems. The first peaks observed for O–H and N–H RDFs were mostly located below 3.1 Å (Supplementary Fig. 39, 40), which indicated the presence of multiple and strong hydrogen bonds among Zn–L···Zn–L units and Zn–L···H_2_O in the mixtures. Meanwhile, the *g*(r) values for the first peaks of O–H and N–H RDFs largely tended to increase with increasing solute content in the system. This tendency suggested that the enhanced intermolecular interactions were achieved by increasing the mass fraction of Zn–L complexes in the mixture, which subsequently promoted the complexes’ aggregation. This was intrinsically in accordance with the analyses of TD-DFT calculations as well as experimental red-shift emissions and prolonged afterglow lifetimes in Zn–L-4/3/2/1 SGs. Taken together, the carboxyl, imidazole, or amino groups of L-His participated in the coordination interaction and hydrogen bonding to promote SG formation, and contributed to the multiple energy levels to promote tunable CPA emissions.

Accordingly, based on our experimental and theoretical investigations, we summarized the following mechanisms for persistent luminescence of Zn–L SGs: (1) strong intra/intermolecular interactions were responsible for the aggregation of the Zn–L complexes and a rigid polymeric microenvironment, to render high-efficiency ISC for achieving various luminous species with tunable excited energy levels and resultant excitation wavelength-dependent CPA emissions; (2) isolated Zn–L complexes only produced microsecond phosphorescence, while the aggregation of the complexes switched on the CPA emissions of the SGs. Then, increasing the aggregation degree of Zn–L complexes caused the narrowing of the energy bandgap, which resulted in solute-dependent CPA emissions of the Zn–L SGs; (3) temperature-dependent CPA emissions may be attributed to the increase of the population of the planar form, which facilitated the narrowing of the energy gap along with the raise of temperature; (4) Zn–L complexes in the SGs absorbed energy from the UV radiation photons could serve as a secondary radiation, while RB acted as an energy reservoir via effective PRET, and the energy was released through orange–red and red CPA emissions of RB-doped SGs. Therefore, strong non-covalent interactions and the aggregation of the complexes, as well as highly efficient ISC and PRET processes laid the foundation for color-tunable CPA in the metal complexes-based SGs.

### Application of luminescent SGs

**Fig. 7:**
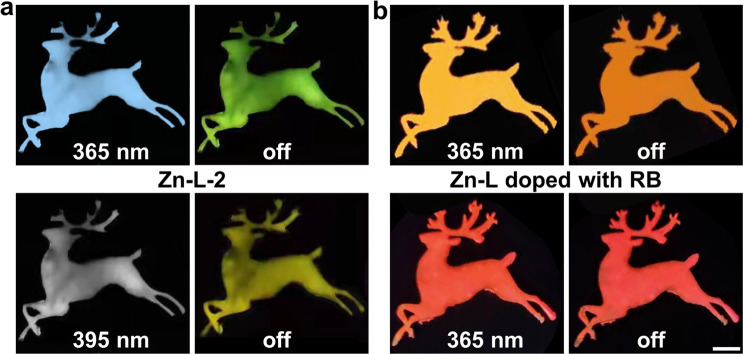
Demonstration of multicolored CPA emissions of SGs for the potential application in displays under ambient conditions. **a** The photos of Zn–L-2 SG in the shape of a deer taken before and after 365/395 nm irradiation turned off in the dark environment. **b** The photos of RB-doped SGs in the shape of deer taken before and after 365 nm irradiation turned off in the dark environment. Scale bar: 0.5 cm.

Due to the good film-forming ability and color-tunable afterglow properties of the as-prepared SGs, their potential application in displays has been explored. During the preparation process, the aqueous solution of Zn–L complexes as well as RB-doped solutions were painted onto the pieces of woods in the shape of deer. After a period of volatilization of the solutions, the emissive patterns with diverse CPA colors were obtained. As shown in Fig. 7a, the patterns painted by the solution of Zn–L complexes glowed green and yellow lasting for a while after ceasing the irradiations of 365 and 395 nm at room temperature, respectively. Notably, the patterns painted by the solutions doped by different molar ratios of RB showed orange–red and red emissions under the irradiation of a UV lamp at 365 nm (Fig. 7b). This was in stark contrast to the blueish-white emission of the pattern painted by the pristine solution of Zn–L complexes. After the UV lamp was turned off, unusual orange–red and red afterglow emissions were observed by the naked eyes. Consequently, these SGs were not only able to be made into large-area films with various shapes (e.g., round sheet and sphere), but also be processed into transparent and multicolored afterglow paintings on diverse substrates. Impressively, once the SG fragments were dissolved in water and then the solution was evaporated for several days, a completely transparent bulk SG was again obtained (Supplementary Fig. 41). This underscored the high recyclability of the SG just through a simple water-mediated route.

## Discussion

In summary, we have fabricated different types of chiral Zn–L complexes-based SGs through an evaporation-induced self-assembly of L-His and Zn^2+^ in water at room temperature. The synergistic effect of the multiple interactions (such as strong metal-ligand and hydrogen bonding interactions) among the L-His, Zn^2+^, and H_2_O, significantly drove the formation of SGs at a macroscopic scale, in which the rigid microenvironment for the chromophores was favorable to boost ultralong RTP. Zn–L SGs presented dynamic tunability regarding solute content-, temperature-, and excitation wavelength-dependent persistent emissions, suggesting the SGs could potentially be further functionalized as chiral optical sensors. Combined experimental and theoretical results confirmed the incorporation of heteroatoms (e.g., nitrogen and oxygen) contributed to multiple energy levels. This also benefitted the formation of different clusters to render efficient ISC for achieving various emission species with tunable excited energy levels. Extraordinary orange–red and red CPA emissions were successfully achieved in dye-doped SGs, affording a PRET efficiency as high as 95.7%. Furthermore, SGs were processed into desired patterns and films with multicolored CPA (decay lifetime up to 356.7 ms, a maximum |*g*_lum_ | value of 9.5 × 10^–3^). It is believed that a variety of advantages, such as simple and environment-friendly synthetic method, easily available building blocks, and facile processing, would endow the color-tunable emissive SGs with high potential in advanced optoelectronic applications.

## Methods

### Materials

Zn(NO_3_)_2_·6H_2_O (Alfa, 99%), L-His (Aladdin, ≥ 99%), D-His (Aladdin, 99%), RB (Aladdin, ≥ 99%), ARS (Matrix, 95 + %), EB (Aladdin, 99.9%), ZnCl_2_ (Alfa, 99.9%), Zn(ClO_4_)_2_·6H_2_O (Alfa, 99.9 %), ZnC_2_O_4_·2H_2_O (Aladdin, ≥ 99 %), Cd(NO_3_)_2_·4H_2_O (Innochem, 99.9%), Eu(NO_3_)_3_·6H_2_O (Innochem, 99.9%), Tb(NO_3_)_3_·5H_2_O (Aladdin, 99.9%), L-Alanine (Innochem, 99%), L-Phenylalanine (Aladdin, 99%), L-Tryptophan (Aladdin, 99%), and L-Cysteine (Aladdin, 97%) and organic solvents (pyridine, methanol, ethanol, acetone and methyl cyanide) (Beijing Oriental Shibo Fine Chemical Co., LTD, ≥ 99.5%) were purchased as indicated and used without further purification. Deionized water was utilized throughout the whole experimental process.

### Synthesis

To study the effect of solute content on the non-covalent interactions and optical behaviors, four samples with different Zn–L mass contents were prepared: Zn–L-1 (98.8 wt%), Zn–L-2 (97.0 wt%), Zn–L-3 (94.7 wt%) and Zn–L-4 (90.5 wt%). To study the effect of the molar ratios of Zn(NO_3_)_2_·6H_2_O to L-His on the stability of the SGs, two samples with basically the same water content as Zn–L-2 SG but different molar ratios of Zn(NO_3_)_2_ to L-His were prepared: Zn–L_1_-2 (1:1) and Zn–L_2.5_-2 (1:2.5); to explore the influence of metal cations and anions on the formation and luminescent characteristics of the SGs, the samples with the water content similar to Zn–L-2 SG and with the same ratio of different types of zinc salts (ZnCl_2_, Zn(ClO_4_)_2_ and ZnC_2_O_4_) and nitrate salts (Cd(NO_3_)_2_, Eu(NO_3_)_3_ and Tb(NO)_3_) to L-His (1:2) were synthesized: Zn-L-Cl, Zn-L-ClO_4_, Zn-L-C_2_O_4_ and Cd-L, Eu-L, Tb-L. Moreover, to obtain orange–red and red CPA SGs, the RB doped Zn–L SGs with nearly-identical Zn–L mass contents (ca. 97.0 wt%) but different ratios of Zn(NO_3_)_2_ and L-His to RB were also synthesized: Zn-L-RB-1 (l:2:2 × 10^–8^) and Zn-L-RB-2 (l:2:6 × 10^–8^). The above SGs as well as corresponding chiral enantiomer SGs based on Zn-D-Histidine (Zn-D) complexes were all synthesized by using the solution method and Zn-D SGs are named by the same way as their enantiomer SGs.

#### Preparation of Zn–L-1/2/3/4 SGs

By slowly evaporating an aqueous solution (10 mL) containing Zn(NO_3_)_2_·6H_2_O (2.97 g) and L-His (3.10 g) in 1:2 molar ratio at room temperature in open air for 5 days and the resultant hydrogel was further dried at room temperature for 10, 20, 25 and 30 days to further remove the solvent, forming Zn–L-4/3/2/1 SGs, respectively. The solute contents (98.8 wt% and 97.0/94.7 wt%) in Zn–L-1 SG and Zn–L-2/3 SGs were determined by the weight (%) based on the TGA curves, respectively, while the solute content (90.5 wt%) of Zn–L-4 SG was determined by the ratio of the obtained weight after heating at 130 °C for 1 h to the weight before heating.

#### Preparation of Zn–L_1_-2 and Zn–L_2.5_-2 SGs

Zn–L_1_-2 and Zn–L_2.5_-2 SGs were obtained by slowly evaporating an aqueous solution (10 mL) containing Zn(NO_3_)_2_·6H_2_O (2.97 g) and L-His (1.55/3.875 g) in 1:1/2.5 molar ratio, respectively, following the process similar to Zn–L-2 SG.

#### Preparation of Zn-L-Cl/ClO_4_/C_2_O_4_ and Cd/Eu/Tb-L SGs

Zn-L-Cl/ClO_4_/C_2_O_4_ and Cd/Eu/Tb-L SGs were prepared in the same way as Zn–L-2 SG, except that Zn(NO_3_)_2_·6H_2_O was separately replaced with other corresponding metal salts (ZnCl_2_, Zn(ClO_4_)_2_·6H_2_O, ZnC_2_O_4_·2H_2_O, Cd(NO_3_)_2_·4H_2_O, Eu(NO_3_)_3_·6H_2_O, and Tb(NO_3_)_3_·5H_2_O).

#### Preparation of dye-doped SGs

RB doped SGs were obtained by slowly evaporating an aqueous solution (10 mL) containing Zn(NO_3_)_2_·6H_2_O (2.97 g), L-His (3.10 g) and RB (4.79 × 10^–7^–28.74 × 10^–7^ g) in the molar ratios of 1:2:1 × 10^–8^–6 × 10^–8^, following the process similar to Zn–L-2 SG. The synthesis processes of ARS and EB doped glassy samples (named as Zn-L-ARS-2 and Zn-L-EB-2, respectively) were the same as that of Zn-L-RB-2 glass, except that RB was replaced by ARS/EB.

#### Preparation of the SGs based on Zn-D complexes

Zn-D-2 and Zn-D-RB-1/2 SGs were prepared by the same method as corresponding SGs based on Zn–L complexes.

#### Preparation of the SGs in the shapes of round sheet and sphere

The SGs in the shapes of round sheet and sphere were prepared by casting corresponding hydrogens into the silicon and plastic modes, respectively, which were then dried at room temperature for 25 days.

#### Preparation of the Zn–L crystal

Zn(NO_3_)_2_·6H_2_O (10 mmol, 2.97 g) and L-His (20 mmol, 3.10 g) with deionized water (12 mL) and pyridine (4 mL) were added to a glass bottle together to form transparent solution, and holes were punched in the film covering the glass bottle to slow down the evaporation of the solvents. After the solution was evaporated at room temperature for about two weeks, the blocky and transparent crystal could be obtained.

### Stiffness measurement

Uniaxial compression tests of the glasses (thickness: ca. 10 mm) were performed by an Instron 3366 electronic universal testing machine (Instron Corporation, Norwood, MA) at ambient condition. The glasses were placed between two metal platens—the lower one fixed, and the upper one made to move downwards at a constant speed of 2 mm s^−1^ to compress the samples up to densification. The three samples of each glass were measured to verify consistency.

### NMR measurement

NMR spectra were recorded at 600 MHz on a SUPERCONDUCTING MAGNET JMTC-600 spectrometer at ambient temperature, and D_2_O was used as the solvent. The spectral data were analyzed by using MestReNova software.

### HR-ESI-MS measurement

HR-ESI-MS measurements were performed on a quadrupole time-of-flight (Q-TOF) mass spectrometer (Q-TOF liquid chromatography/mass spectrometry (LC/MS) 6540 series, Agilent Technologies, Santa Clara, CA) coupled with electrospray ionization (ESI). m/z: calculated for Zn(L-His-H)_2_ (C_12_H_17_N_6_O_4_Zn^+^) ([M + H]^+^): 373.0597, found, 373.0733, m/z: calculated for L-His (C_6_H_10_N_3_O_2_^+^) ([M + H]^+^): 156.0768, found 156.0824.

### XPS measurement

The XPS data of Zn–L crystal and glasses were performed on a Thermo Scientific Escalab 250Xi spectrometer, using a monochromatic Al Kα source. All spectra were calibrated using inorganic carbon peak (284.80 eV) as a reference.

### FT-IR measurement

FT-IR spectra of the SGs were measured on a Bruker TENSOR 27 infrared spectrophotometer. Each spectrum was recorded by performing 32 scans between 4000 and 400 cm^−1^. Before measurement, the as-prepared samples were mixed with potassium bromide (KBr) and pressed as pellets.

### Elemental analysis

Elemental analyses (C, H, and N) were performed on a Vario EL elemental analyzer.

### Single-crystal XRD measurement

Single-crystal X-ray diffraction data of the sample were collected at 100 K on Bruker SMART APEX CCD diffractometer employing monochromatized Cu Kα radiation (λ = 1.54184 Å).

### Thermal analysis

DSC thermograms of the SGs, L-His powder and Zn–L crystal were performed on the METTLER TOLEDO DSC 1 calorimeter under N_2_ atmosphere with a heating rate of 5 °C min^−1^. TGA analyses of them were measured on NETZSCH STA449 F5 Thermogravimetric Analyzer with a heating rate of 5 °C min^−1^ under N_2_ atmosphere.

### UV-Vis-NIR transmittance/absorption measurement

The transmittance spectra of Zn–L/D-2 SGs and Zn-L-RB-1/2 SGs were carried out on a SPECORD200 spectrophotometer by positioning the glasses (ca. 5 mm thick) perpendicular to the incident beam. The absorption spectra of Zn–L and L-His solutions were performed on a Shimadzu UV-3600 spectrophotometer. The UV-Vis absorption spectrum of RB powder was carried out on a SPECORD200 spectrophotometer and BaSO_4_ as the reference.

### PXRD measurement

PXRD data of the SGs and Zn–L crystal were performed on a Rigaku Ultima-IV automated diffraction system with Cu Kα radiation (λ = 1.5406 Å), and the operating power was 40 kV, 30 mA. The measurements were made in a 2θ range of 10°–50° at ambient temperature with a step of 0.02° (2θ) as well as scan speed of 5° min^−1^. The simulated PXRD pattern of Zn–L crystal was generated using Mercury software.

### Fluorescence microscope observations

The photographs of the glasses under the irradiation were taken with an OLYMPUS IX71 fluorescence microscope (Tokyo, Japan).

### TEM measurement

The TEM images and EDS maps were performed on Talos F200S operated at 200 kV. To prepare the sample for TEM analysis, Zn–L-1 SG (1 mg) was dissolved into 1 mL water under sonication for 1 h till the clear solution was obtained and then 4–5 drops were casted on copper mesh coated with an ultrathin carbon film and dried in air for 3 days.

### PL measurement

All the relevant photoluminescence tests were recorded on an Edinburgh FLS-980 fluorescence spectrometer with a xenon arc lamp (Xe900) and a microsecond flash-lamp (µF900). The PL lifetimes (τ) of the samples were obtained by fitting the decay curve with a multi-exponential decay function of I(t) = A_1_exp(−t/τ_1_) + A_2_exp(−t/τ_2_) +…+ A_i_exp(−t/τ_i_), where A_i_ and τ_i_ represent the amplitudes and lifetimes of the individual components for multiexponential decay profiles. The absolute PL quantum yields of the SGs at room temperature were reckoned by using an integrating sphere (F-M101, Edinburgh) accessory in FLS-980 fluorescence spectrometer.

### CD and CPL signals measurement

The CD and CPL spectra of the samples were recorded directly on a JASCO J-1500 CD spectrometer, and a JASCO CPL-200 spectrophotometer, respectively. Before measurement of the CD and CPL spectra, the glassy samples were fixed to the sample holder, and the excited light source passed directly through the transparent glasses with the thickness of no more than 3 mm; the powder and crystal samples were sandwiched between two pieces of transparent quartz glasses, the ends of which were taped together and secured to the sample holder.

### Theoretical calculations

Density functional theory (DFT) and time-dependent DFT (TD-DFT) simulations were performed with Gaussian 09 package^66^. All the computational models were extracted from the structures of the single-crystals without further geometry optimization. The electronic transitions, excitation energy of the singlet states (S_n_) and the triplet state (T_n_), and electrostatic potential were calculated by TD-DFT method of B3LYP/6-311 + G(d) level based on the monomer and selected aggregates extracted from the single-crystals. The ESP distribution maps were visualized through VMD software.

Molecular dynamic (MD) simulations were carried out by using Material Studio (version 19.1). The interactions in Zn-L–H_2_O systems were investigated by the RDFs, and the RDF results were directly obtained from the final MD results. The structures of Zn–L complex and water molecule were optimized under specified conditions (force field: COMPASS, Charges: Forcefield assigned, Quality: Fine, Summation method: Atom based)^67^. Total 500 molecules were placed into a cubic solution box based on the real composition. Herein, the mass fractions of Zn–L complexes in three investigated boxes, were at roughly 94.7, 97.0 and 98.8 wt%, respectively. Subsequently, the solution environment was optimized for ensuring energy minimization. Finally, the dynamic calculation was performed with total simulation time of 300 ps (ensemble: NVT, thermostatic: nose, integration time step: 1 fs)^65^.

The supplementary videos were recorded by a Nikon D3500 camera in the dark and under ambient conditions.

### Reporting summary

Further information on research design is available in the Nature Portfolio Reporting Summary linked to this article.

## Supplementary information

Supplementary Information

Description of Additional Supplementary Files

Supplementary Data 1

Supplementary Movie 1

Supplementary Movie 2

Supplementary Movie 3

Supplementary Movie 4

Reporting Summary

## Data Availability

All data are available from the authors upon request.
