# Supplementary material for: Supramolecular glasses with color-tunable circularly polarized afterglow through evaporation-induced self-assembly of chiral metal–organic complexes
Source: Nat Commun. 2023 Mar 24;14:1654. doi: 10.1038/s41467-023-37331-0 (PMC10039082; doi:10.1038/s41467-023-37331-0)
Supplement: Supplementary file 1 — Supplementary Information [file 41467_2023_37331_MOESM1_ESM.pdf]

Supplementary Information

Supramolecular glasses with color-tunable circularly polarized afterglow through evaporation-induced self-assembly of chiral metal–organic complexes

Fei Nie[1], Ke-Zhi Wang[1], and Dongpeng Yan[1]*

[1]Beijing Key Laboratory of Energy Conversion and Storage Materials, College of Chemistry, Key Laboratory of Radiopharmaceuticals, Ministry of Education, Beijing Normal University, Beijing 100875, P. R. China.

Correspondence and requests for materials should be addressed to D. Y. (email: yandp@bnu.edu.cn).

Supplementary Note 1: The key to the fabrication of the SGs

In the primary process of the volatilization, the aqueous solution of the metal-ligand complexes was just a free flowing fluid with a low viscosity and consistent structure. However, as the volatilization proceeded, the solution gradually became viscous (as shown by Supplementary Figure 1), just analogous to a supercooled glass-forming liquid[1, 2]. It was relatively difficult for the complexes to form crystals in the solution with high viscosity, because the nucleation and crystal growth in these viscous liquids were both mobility-limited[1]. As the free solvent continued evaporating, the more viscous solution "froze" into a glassy state with microscopic inhomogeneity. Hence, we speculate that the key to the fabrication of the supramolecular glass in this work may take the advantage of viscous retardation during nucleation and crystallization upon the evaporation of solution.

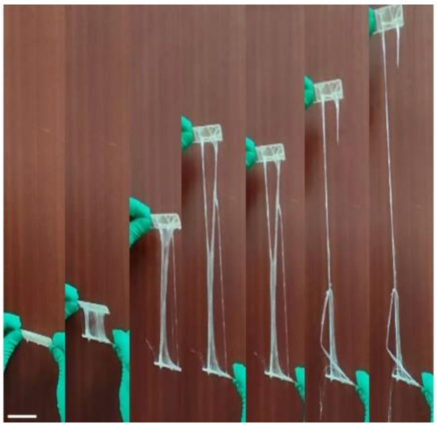

Supplementary Figure 1 | The viscoelastic property of Zn-L gel. Scale bar: 15 mm.

Supplementary Note 2: The results of quasi-static compression tests for the SGs

Results of the quasi-static compression tests manifested that the initial yield stress parameters of Zn-L-2 and Zn-L-RB-1/2 SGs were 1.14, 1.00, 0.52 MPa, respectively, which were comparable to that of the 3D-printed sample made from polylactic acid in our daily life[3], indicating the high stiffness of the glasses[4, 5]. The ductile fracture of SGs enabled the supramolecular network structures to be compressed continuously, rather than showed abrupt collapse at a small strain, especially as those of many materials fabricated using crystallization strategy. The decreased compressive strength of the glassy samples by the doping of RB may be caused by the coordination of doped RB to zinc(II) ion[6], which decreased the network connectivity in the SG matrixes.

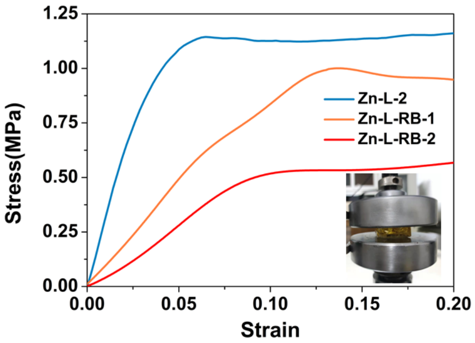

Supplementary Figure 2 | Engineering stress-strain curves from compression tests on the glasses.

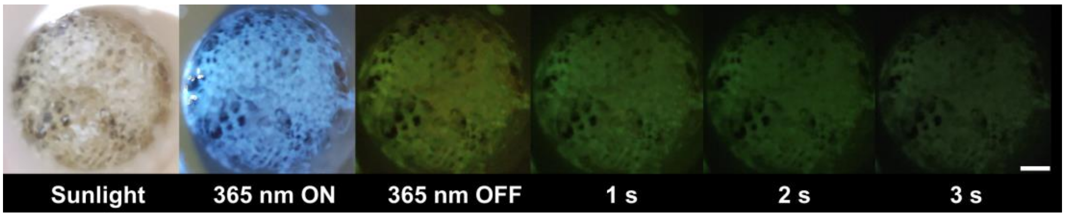

Supplementary Figure 3 | Images of the glassy sample prepared at 90 °C were taken before and after 365 nm irradiation turned off under ambient conditions. Scale bar: 25 mm.

Supplementary Note 3: The results of ^{1}H NMR spectra for Zn-L glass, crystal and gel

The peak positions of the protons in the complexes present in the three forms (glass, crystal and gel) were nearly identical, indicating the consistency of the structure of Zn-L complex in the three types of materials.

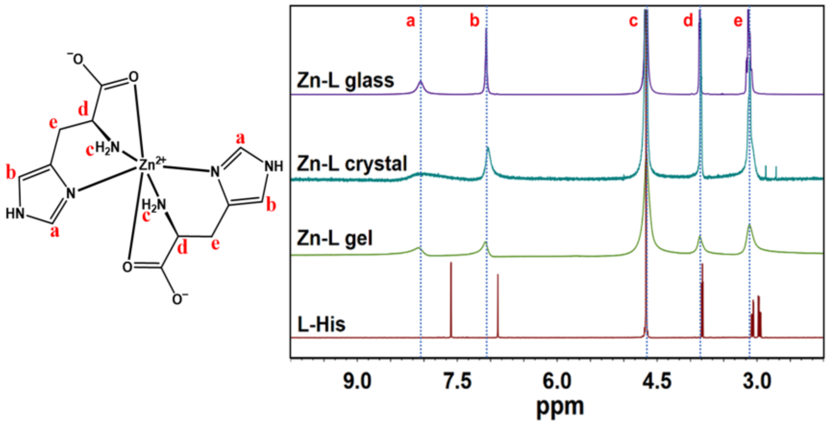

Supplementary Figure 4 | The ^{1}H NMR spectra of L-His powder as well as Zn-L gel, crystal and glass.

Supplementary Note 4: The purity illustration of the L-His powder used in this work

We have used L-His powders purchased from different manufacturers (Aladdin (purity, ≥99%); Macklin (purity, 99.5%); Innochem (purity, 99.0%); Tci (purity, ≥99.0%); Xiya (purity, ≥99.5%); HWG (purity,

99.0%)) in our experiment. The high purity and structure of the commercial L-His were verified by NMR/HR-ESI-MS/FT-IR measurements operated by the manufacturers. In addition, these L-His powders were prepared by using different processes, as well as different animal or non-animal sources. As such, the little residues in these L-His powders may vary in compositions and contents. Significantly, the supramolecular glasses prepared from these L-His powders exhibited almost identical photoluminescence performance (Supplementary Figure 5b), proving that the little residues had negligible effect on structures/properties of the glasses.

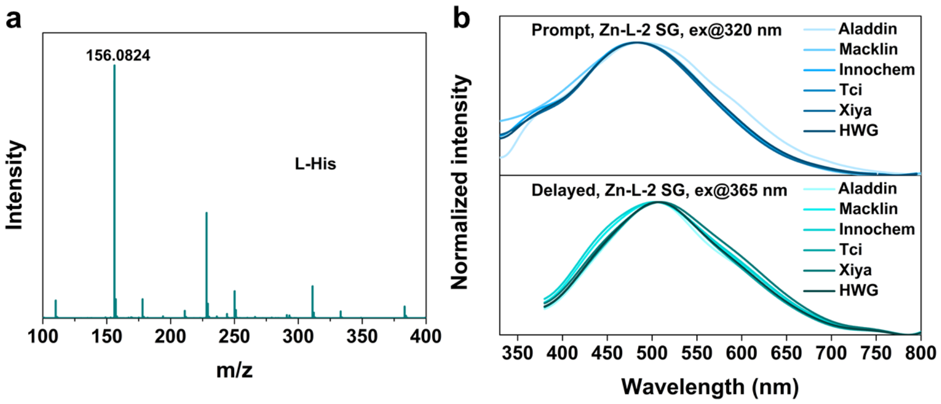

Supplementary Figure 5 | The purity proof of the L-His powder. (a) The HR-ESI-MS spectrum of L-His powder. (b) The prompt and delayed PL spectra of Zn-L-2 SG prepared from L-His powders purchased from different manufacturers.

Supplementary Note 5: The XPS results for Zn-L glass, crystal and gel

The XPS bands of Zn 2*p* (binding energies at 1022.0 and 1045.1 eV) and N 1*s* (binding energy at 399.5 eV) orbitals for Zn-L glass, crystal and gel were nearly identical, implying the similarity of chemical environment and electronic structure of Zn-L complex in the three types of materials[7]. This seemed to further confirm the integrity of the chemical structure of Zn-L complex during volatilization. The bands in N 1*s*-XPS spectra at 405.9 eV were ascribed to the nitrate ions, which existed as impurities in the glass network.

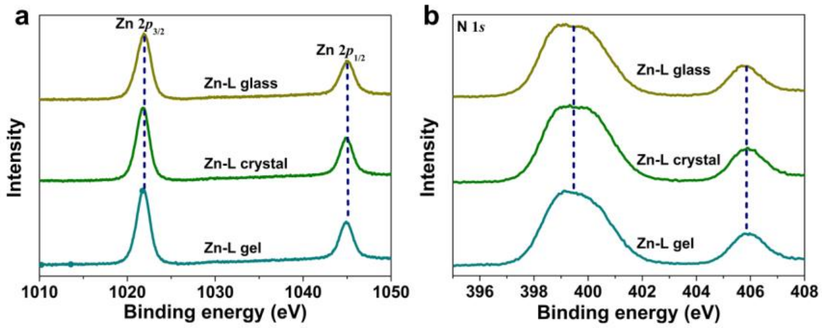

Supplementary Figure 6 | XPS results for Zn-L gel, crystal and glass.

Supplementary Note 6: The results of FT-IR spectra for the SGs, L-His, $Zn(NO_3)_2$, and Zn-L crystal

The observable peaks in FT-IR spectra of Zn-L SGs at 826 cm^{-1} were attributed to the vibration of nitrate groups (Supplementary Figure 7a). For Zn-L-RB-1/2 SGs (Supplementary Figure 7b), the appearance of two new Zn-N peaks at 532 and 649 cm^{-1} and the red shift of the symmetric C=O stretching of the carboxylate group (1506 cm^{-1}) relative to that (1587 cm^{-1}) for L-His powder implied the coordination of amino, imidazole and carboxylate groups with Zn^{2+}, respectively. Additionally, for Zn-L crystal (Supplementary Figure 7c), the peaks at 532, 649 and 1504 cm^{-1} indicated the coordination of these three groups with Zn^{2+}.

The FT-IR spectra of Zn-L-2/3 and Zn-L-RB-1/2 SGs showed absorption peaks similar to those of Zn-L-1 SG in the range of 4000–400 cm^{-1}, indicating that there were basically identical coordination structures in these SGs. That is to say, the structural integrity of Zn-L complexes in the glass matrix could be confirmed during the volatilization process and even doped with RB.

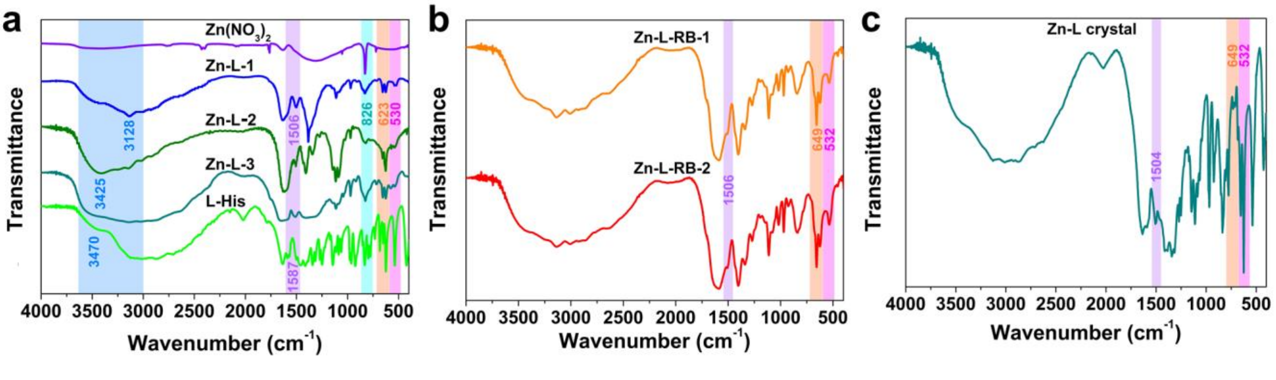

Supplementary Figure 7 | The FT-IR spectra results. (a) FT-IR results for Zn-L-1/2/3 glasses, L-His powder, $Zn(NO_3)_2 \cdot 6H_2O$, (b) Zn-L-RB-1/2 glasses and (c) Zn-L crystal.

Supplementary Note 7: The PXRD pattern results of Zn-L crystal

PXRD pattern of Zn-L crystal could match well with the simulated results.

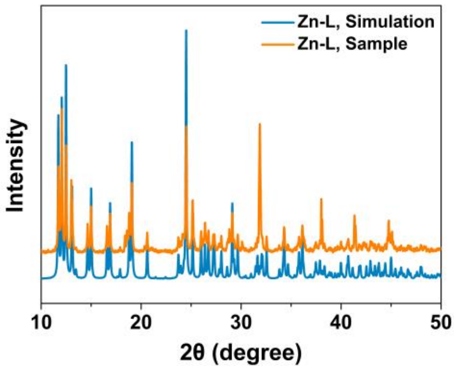

Supplementary Figure 8 | PXRD pattern of Zn-L crystal and the simulated crystal structure data.

Supplementary Note 8: The possible configurations of the Zn-L complex isomers in the SGs

The possible configurations of the isomers (i.e., *trans(*N_π*)*-, *trans(O)*-, and *trans(*N_H*)*-Zn(L-His)$_2$)[8] of Zn-L complex in the SGs could be assigned favorably from their characteristic CD, NMR and FT-IR spectra[8–12]. Considering that, the very close CD, ^{1}H NMR, and FT-IR spectra between the SGs and the single crystal form confirmed the structure of Zn-L complex in the SG was highly similar to that in the crystalline state (Fig. 2b, 5d and Supplementary Fig. 4, 7 and 29a).

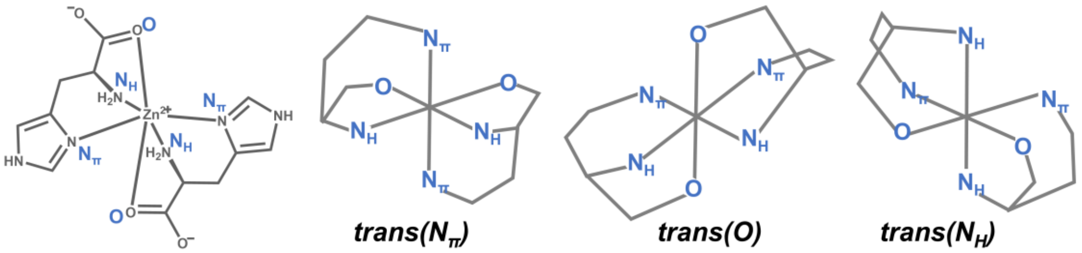

Supplementary Figure 9 | Three possible geometrical isomers of the Zn-L complex in the SGs.

Supplementary Note 9: The DSC results of the SGs

The appearance of T_g in the DSC curves suggested the formation of the SGs. The observable T_g (40.1 and 32.0 °C) in the DSC curves of Zn-L-RB-1/2 SGs verified a small amount of RB could be successfully integrated into the Zn-L complexes-based glass matrixes (Supplementary Figure 10a). Additionally, the T_g values of Zn-L_1-2 (63.8 °C) and Zn-$L_{2.5}$-2 (42.2 °C) SGs could be observed (Supplementary Figure 10b).

Considering that the presence of non-covalent interactions and cross-linked structure had the ability to enhance T_g by decreasing free volume[13, 14], the anions (NO_3^-, CI^-, CIO_4^{2-}, $C_2O_4^{2-}$) with different electronegativity and as impurities in the glass network, probably exerted certain influence on the metal-ligand interaction and crosslinked structure, and thus affected the T_g of the corresponding glasses (Supplementary Figure 10c)[15].

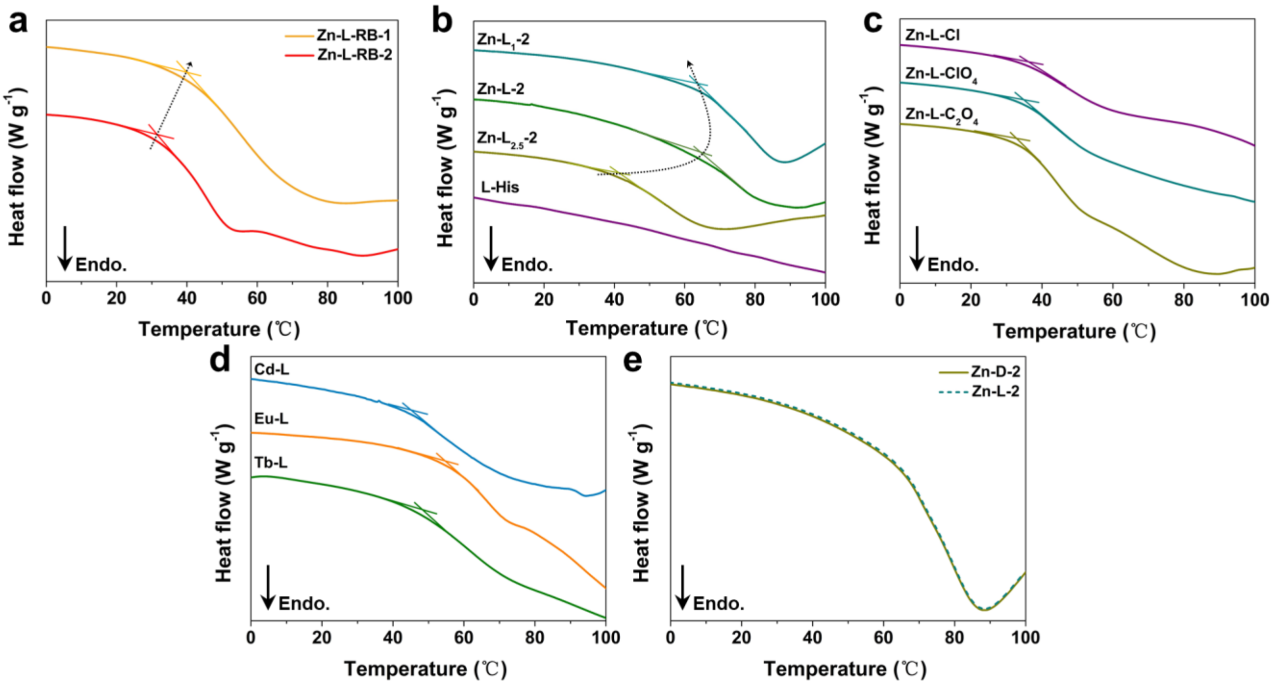

Supplementary Figure 10 | The DSC curves of the SGs. (a) DSC curves of Zn-L-RB-1/2, (b) Zn-L_1-2, Zn-L-2, Zn-$L_{2.5}$-2 SGs and L-His powder. (c) DSC curves of Zn-L-Cl/ClO_4/C_2O_4, (d) Cd/Eu/Tb-L and (e) Zn-L/D-2 SGs.

Supplementary Note 10: The TGA results of the SGs and Zn-L crystal

As shown in Supplementary Figure 11a, the overall weight loss of water in the range of 25–150 °C was 1.2% for Zn-L-1 SG, while the overall weight losses in the range of 25–130 °C were 3%, 5.3% for Zn-L-2/3 SGs, respectively. The initial degradation temperature of Zn-L-3 SG was below 225 °C and its specific value could not be determined by the curve.

In the TGA trace of Zn-L-1 SG, the continuous weight loss in the range of 25–150 °C was mainly related with the gradual loss of free water molecules and hydrogen-bonded water molecules. In contrast, the almost complete loss of the structured water occurred at 130 °C for Zn-L-2 SG. These reflected that there existed stronger hydrogen-bonding between solvent water molecule and the complex in Zn-L-1 SG compared to that in Zn-L-2 SG. Notably, as the volatilization proceeded, for Zn-L-3/2/1 SGs, relatively more structured water molecules and a smaller proportion of free water molecules existed in the glass matrix, which resulted in an increase of the network connectivity in light of lowering of the probability for free water molecules to intervene in the connection between the neighbored supramolecular structural units. Moreover, Zn-L-1/2 SGs showed the initial degradation temperatures at approximately 233 and 225 °C, respectively, which were higher than that of Zn-L-3 SG. Based on these results, the phenomenon that the initial degradation temperature

increased with the increase of solute mass fraction in Zn-L-1/2/3 SGs indicated that the enhanced non-bonded interactions were conducive to the construction of a highly stable polymeric structure.

The initial degradation temperature of Zn-L-2 SG was higher than those of the dye doped samples (Zn-L-RB-1/2 SGs) (Supplementary Figure 11b), probably because the coordination between trace RB and Zn^{2+} was not conducive to the structurally thermal stability of the SGs[6].

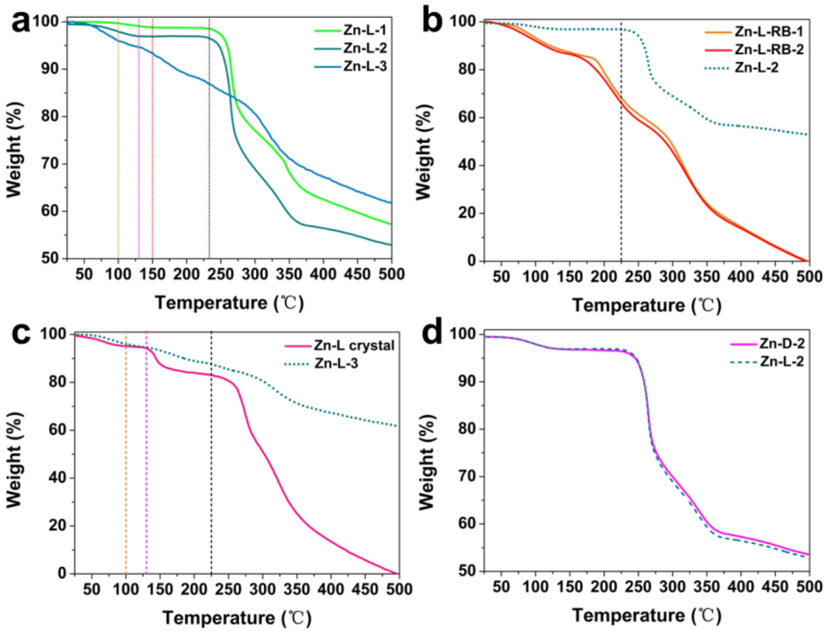

Supplementary Figure 11 | The TGA results of the SGs and Zn-L crystal. (a) TGA curves of Zn-l-1/2/3 SGs, (b) Zn-L-RB-1/2 SGs, (c) Zn-L crystal and (d) Zn-D-2 SG. The orange, violet, red and black lines in these figures are located at 100, 130, 150 and 225 °C, respectively.

Supplementary Note 11: The UV-Vis-NIR transmittance spectra results for Zn-L-RB-1/2 SGs

The Zn-L-RB-1/2 glasses were highly transparent (above *ca.* 70%) in the visible and NIR ranges (gray areas). In the wavelength range of 200–1800 nm, the as-synthesized glasses showed several absorption bands, which were attributed to Zn-L complexes (cyan areas), RB (pink area) and water (blue areas), respectively.

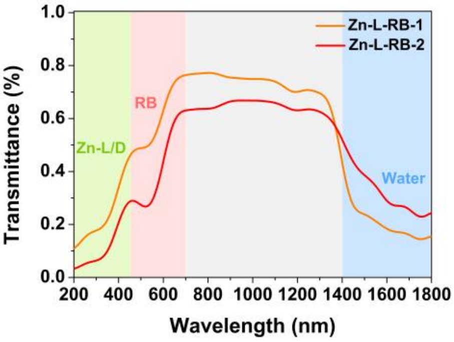

Supplementary Figure 12 | UV-Vis-NIR transmittance spectra results for Zn-L-RB-1/2 glasses.

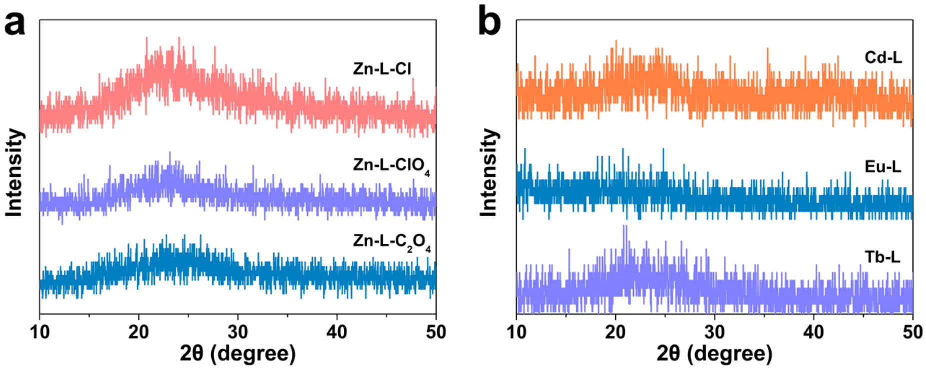

Supplementary Figure 13 | The PXRD patterns of the SGs. (a) PXRD patterns of Zn-L-Cl/ClO_4/C_2O_4 and (b) Cd/Eu/Tb-L SGs.

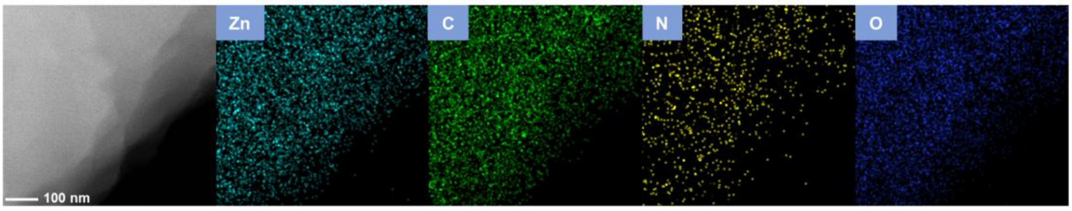

Supplementary Figure 14 | TEM image of the Zn-L SG and its EDS mapping showed Zn, C, N, and O, with a homogeneous distribution of elements.

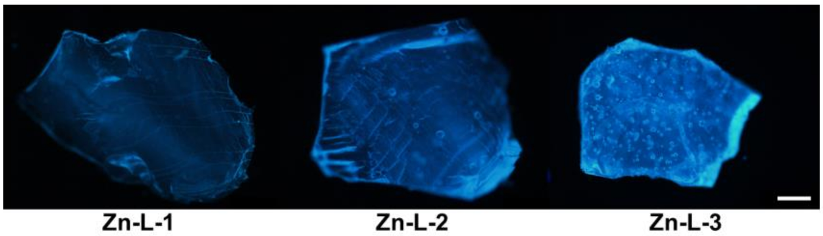

Supplementary Figure 15 | The fluorescence microscopy images of as-prepared Zn-L-1/2/3 SGs taken under UV irradiation. Scale bar: 50 μm.

Supplementary Note 12: The analyses about one band in the prompt PL spectrum of L-His powder

Expectantly, there existed only one band in the prompt PL spectrum of L-His powder (Supplementary Figure 16a). Hence, we observed the phenomenon of 'no separation' between fluorescence and phosphorescence bands at ambient conditions in the prompt PL spectra of Zn/Cd complexes-based SGs (Supplementary Figure 18a, 19a, 21a, 24a, 25a), which may be derived from the photophysical properties of L-His fractions as luminophores in the glass matrixes.

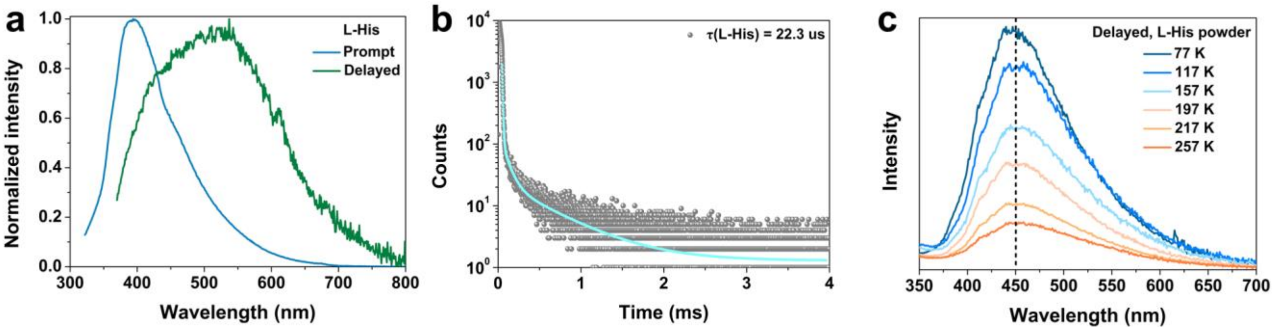

Supplementary Figure 16 | The luminous behaviors of L-His powder. (a) Prompt and delayed PL spectra of L-His powder excited by 305 and 365 nm, respectively, and (b) its phosphorescence lifetime decay curve excited by 365 nm. (c) The delayed PL spectra of L-His powder excited by 330 nm at different temperatures.

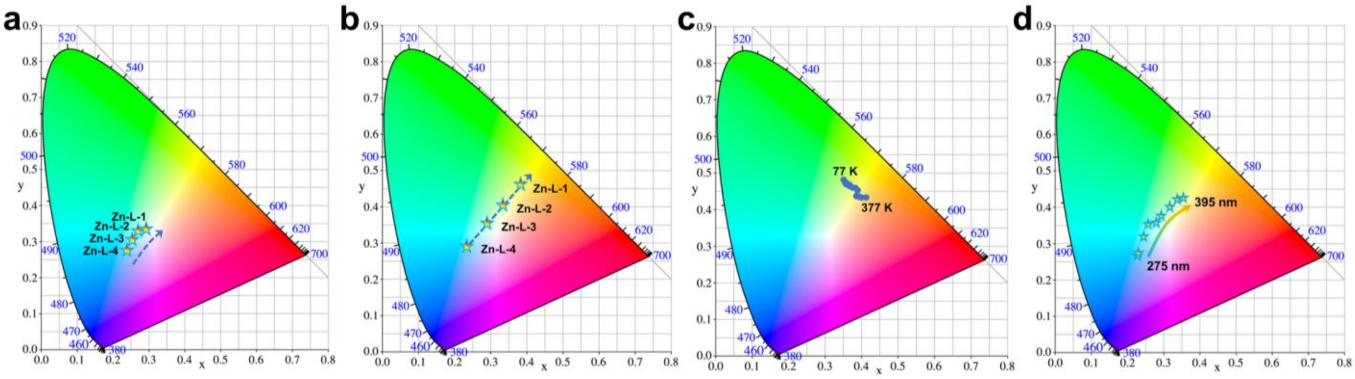

Supplementary Figure 17 | The emissive positions in the chromaticity coordinates diagrams of the SGs. (a) The corresponding positions of the different emissions in the chromaticity coordinates diagrams of Zn-L-1/2/3/4 SGs under prompt and (b) delayed modes. (c) The corresponding positions of the different phosphorescence emissions in the chromaticity coordinates diagrams of Zn-L-2 SG excited by 365 nm at different temperatures and (d) excited by different wavelengths at room temperature.

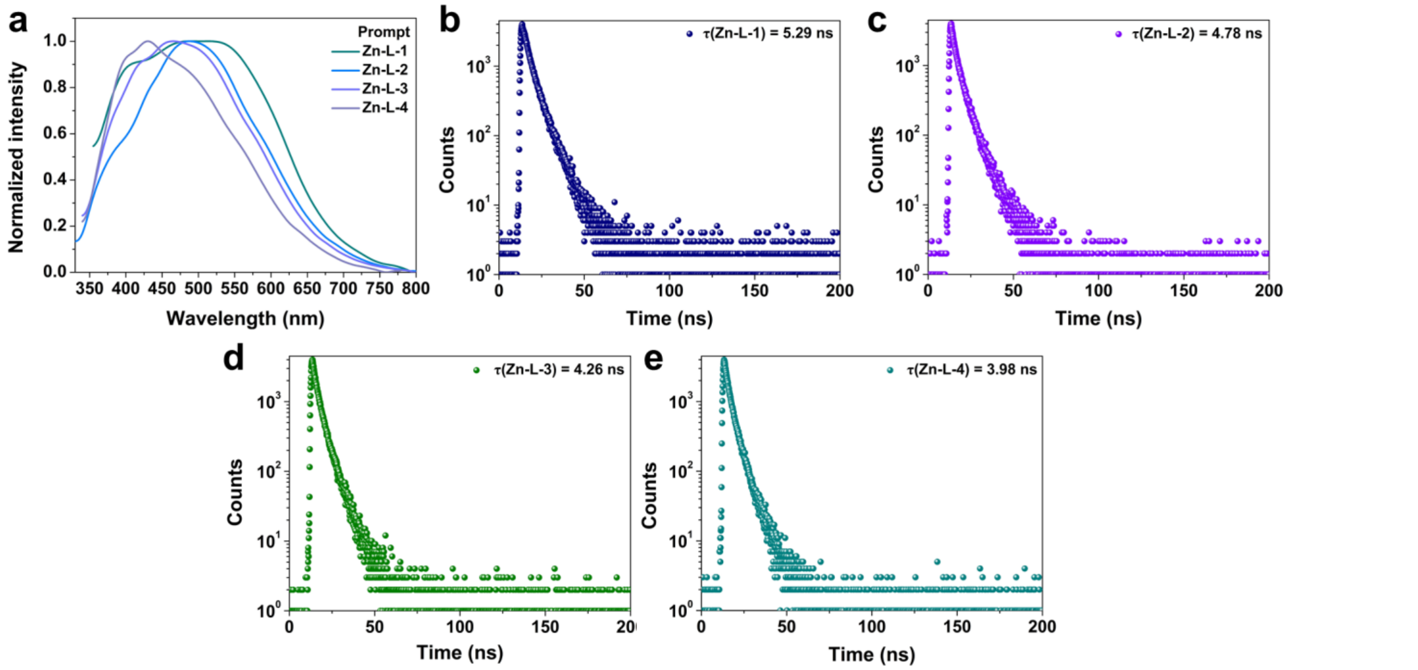

Supplementary Figure 18 | Prompt PL spectra and the fluorescence decay profiles of the SGs. (a) Prompt PL spectra of Zn-L-1/2/3/4 SGs excited by 320 nm and (b–e) their fluorescence decay profiles at 500, 489, 466 and 430 nm at room temperature, respectively.

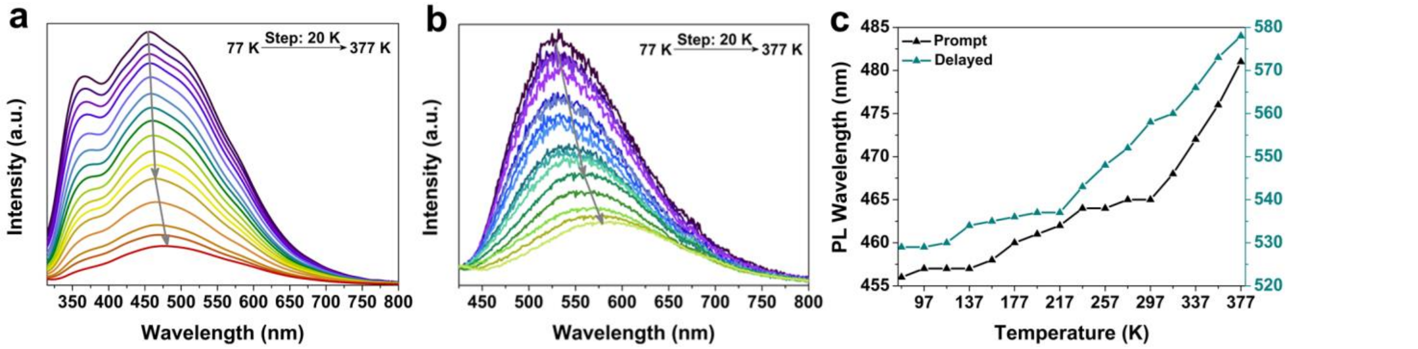

Supplementary Figure 19 | Temperature-dependent luminescence properties of Zn-L-2 SG. (a, b) Temperature-dependent prompt and delayed PL spectra of Zn-L-2 SG excited by 320 and 365 nm, respectively. (c) The relationship between the temperature and emission wavelengths (prompt and delayed modes) of Zn-L-2 SG.

Supplementary Note 13: The results of excitation-dependent phosphorescence lifetimes for Zn-L-2 SGs

The excitation-dependent phosphorescence lifetimes of Zn-L-2 glass further confirmed the WDP property of the SGs.

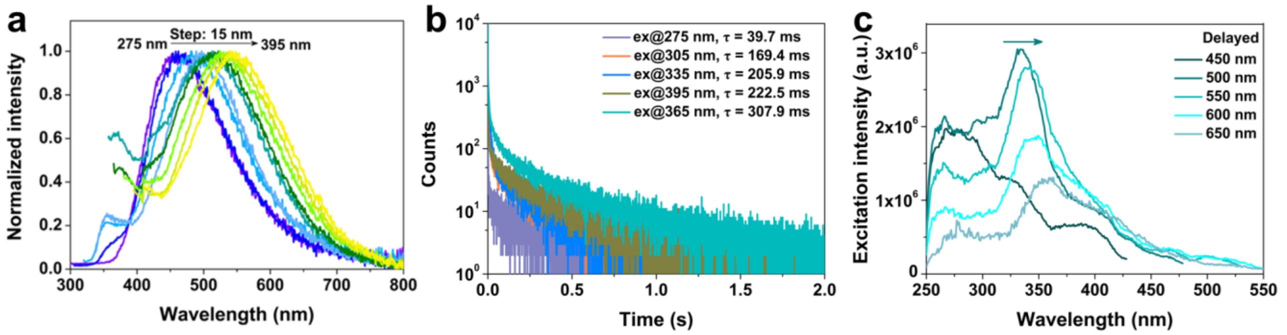

Supplementary Figure 20 | Excitation wavelength-dependent emissions of Zn-L-2 SG. (a) Excitation wavelength-dependent phosphorescence emissions of Zn-L-2 SG. (b) Phosphorescence decay curves of Zn-L-2 SG excited by different wavelengths. (c) Delayed PL excitation spectra of Zn-L-2 SG at different emission wavelengths.

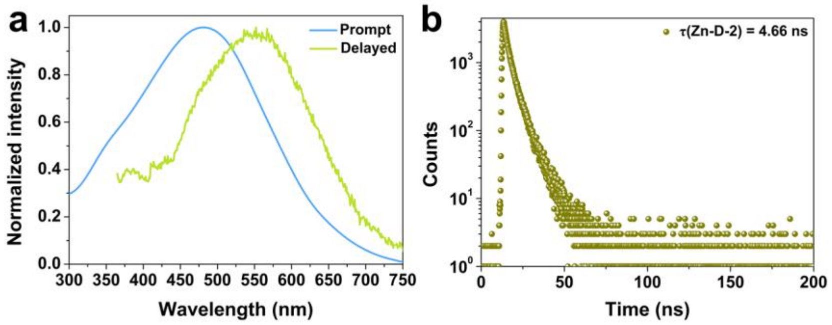

Supplementary Figure 21 | The luminescent behaviors of Zn-D-2 SG. (a) Prompt and delayed PL spectra of Zn-D-2 SG excited by 320 and 365 nm, respectively, and (b) its fluorescence decay curve at 483 nm.

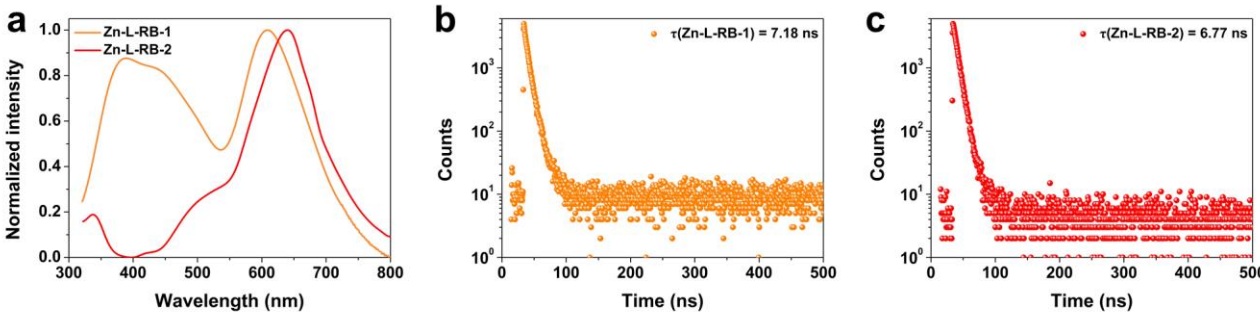

Supplementary Figure 22 | The photophysical properties of Zn-L-RB-1/2 SGs. (a) Prompt PL spectra of Zn-L-RB-1/2 SGs excited by 305 nm and (b, c) their fluorescence decay profiles at 609 and 640 nm.

Supplementary Note 14: The luminescent behaviors of Zn-L-ARS/EB-2 glassy samples

In their prompt and delayed PL spectra, Zn-L-ARS/EB-2 glassy samples both exhibited two emission bands: the short wavelength and long wavelength spectral bands originated from the emissions of the Zn-L complexes and ARS/EB, respectively (Supplementary Figure 23a). Moreover, their decay curves exhibited ultralong

lifetimes of 157.8 ms (at 588 nm) and 102.2 ms (at 675 nm), respectively (Supplementary Figure 23b). These results corroborated the successful achievement of PRET from the Zn-L complexes to the dyes, benefiting from the large overlap between the RTP spectrum of Zn-L complexes and the absorption spectrum of the dyes molecules as well[16, 17]. Hence, it was reasonable to conclude that the large spectral overlap was a prerequisite for the PRET process.

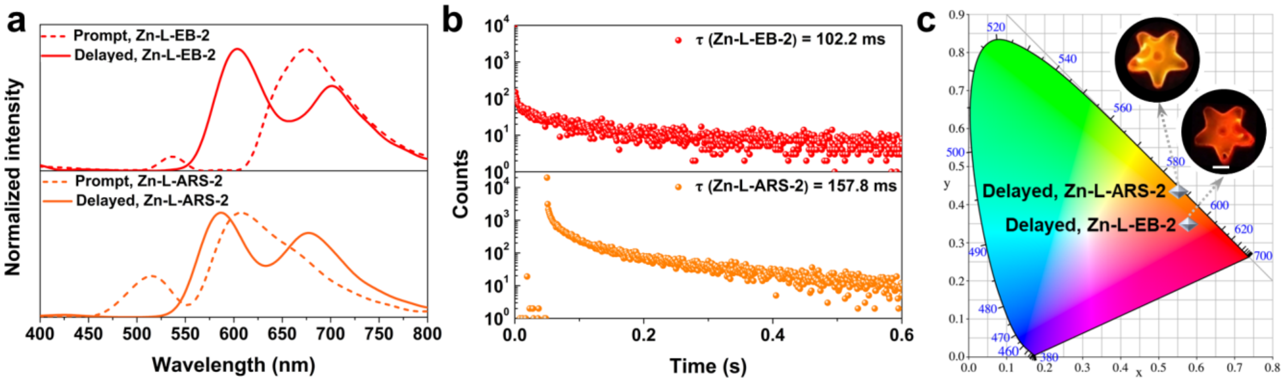

Supplementary Figure 23 | The PL properties of Zn-L-ARS/EB-2 glassy samples. (a) Prompt and delayed PL spectra of Zn-L-ARS/EB-2 glassy samples excited by 320 and 365 nm, respectively, and (b) their phosphorescence decay curves at 588 and 675 nm, respectively. (c) Corresponding positions of the emissions in the chromaticity coordinates diagram of Zn-L-ARS/EB-2 glassy samples under delayed modes (inset: their afterglow photographs excited by 365 nm UV lamp). Scale bar: 50 mm.

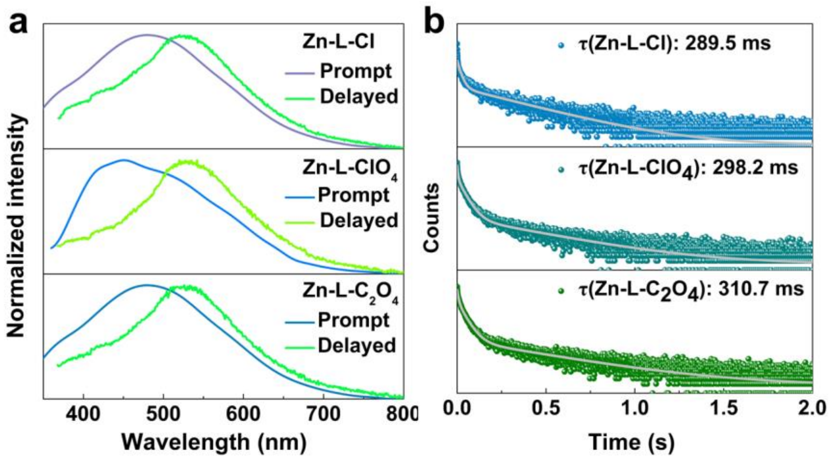

Supplementary Figure 24 | The PL properties of Zn-L-Cl/ClO$_4$/C$_2$O$_4$ SGs. (a) Prompt and delayed PL spectra of Zn-L-Cl/ClO$_4$/C$_2$O$_4$ SGs excited by 320 and 350 nm, respectively, and (b) corresponding phosphorescence decay curves at 530 nm.

Supplementary Note 15: The results of the phosphorescent spectra for Eu/Tb-L SGs

For the phosphorescence spectra of Eu/Tb-L SGs (Supplementary Figure 25c), the sharp lines centered at 594, 621, 654, 700 nm and 490, 545, 585, 622 nm could be ascribed to $^5D_0 \rightarrow {}^7F_J$ (J = 1–4) transitions of Eu^{3+} and $^5D_4 \rightarrow 7F_J$ (J = 3–6) transitions of Tb^{3+}, respectively.

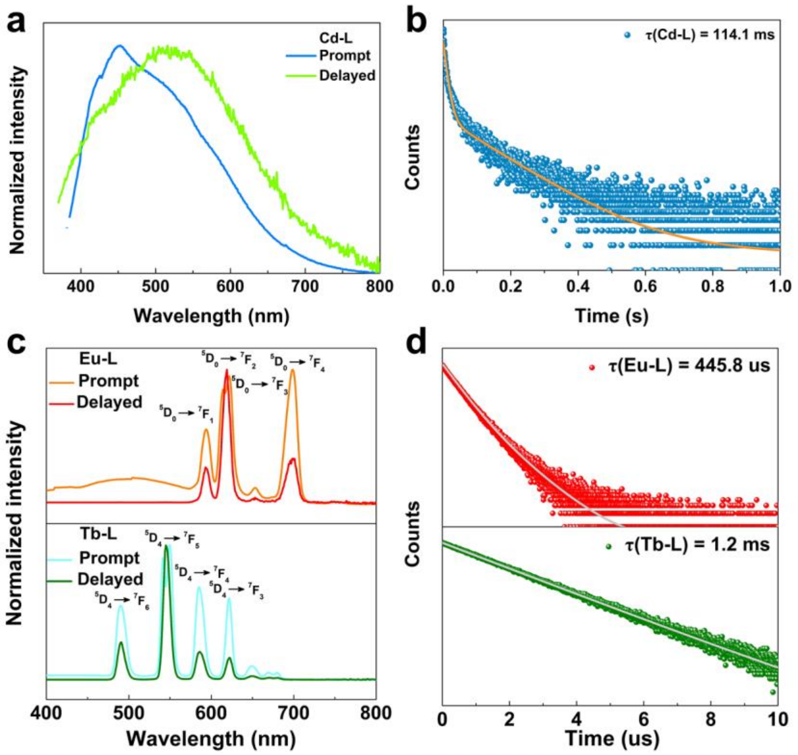

Supplementary Figure 25 | The photophysical properties of Cd/Eu/Tb-L SGs. (a) Prompt and delayed PL spectra of Cd-L SG excited by 320 and 350 nm, respectively, and (b) its phosphorescence decay curve at 522 nm. (c) Prompt and delayed PL spectra of Eu/Tb-L SGs excited by 350 nm and (d) their phosphorescence decay curves at 621 and 546 nm, respectively.

Supplementary Note 16: The luminous behaviors of Zn-L crystal

The prompt PL spectrum of Zn-L crystal exhibited a sharp peak in the ultraviolet region (λ_{em} = 360 nm) and one shoulder peak in the range of 440–600 nm (Supplementary Figure 26a). Its delayed PL spectrum (λ_{em} = 509 nm) nearly overlapped with the region of the shoulder peak in the prompt one, manifesting that the luminescence of Zn-L crystal featured both blue fluorescence and green phosphorescence characteristics.

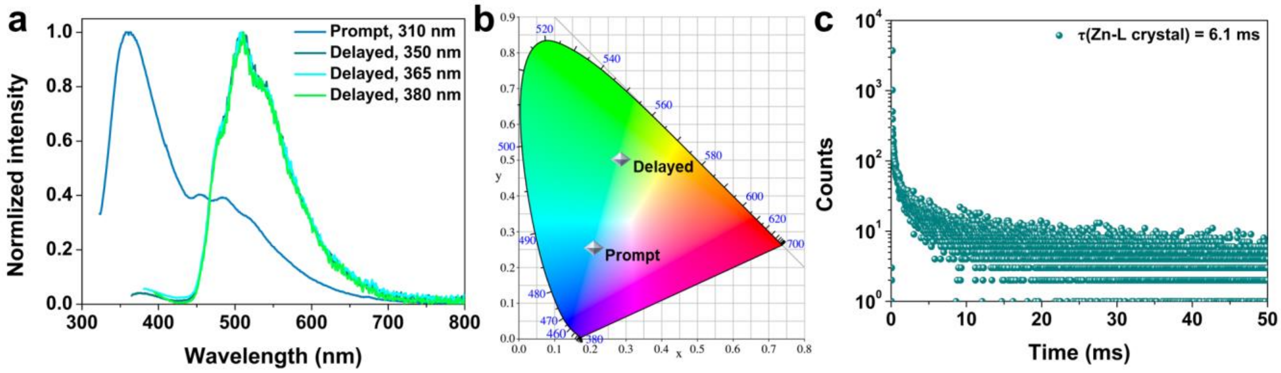

Supplementary Figure 26 | The PL characteristics of Zn-L crystal. (a) Prompt PL spectrum of Zn-L crystal excited by 310 nm and its delayed PL spectra excited by different wavelengths. (b) The corresponding positions of the emissions in the chromaticity coordinates diagram of Zn-L crystal under prompt and delayed modes. (c) Phosphorescence decay curve of Zn-L crystal at 508 nm when excited by 365 nm.

Supplementary Note 17: The analyses about the g_{lum} achieved in the SGs

Theoretically, g_{lum} can be calculated according to the equation: $g_{\mathrm{lum}} = 4(|u||m|\cos\theta_{\mu,m})/(|u|^2+|m|^2)$[18, 19], where u and m represent the electric and magnetic transition dipole moments, respectively, and $\theta_{\mu,m}$ represents the angle between these two dipole moments. $|g_{\mathrm{lum}}|$ reaches the maximum value of 2 when u and m are equal to each other in length and oriented in either the parallel or antiparallel direction. However, due to fact that the length of m is generally much smaller than that of u in most of small organic molecules, the denominator in the equation is dominated by $|u|^2$, which generates the simplified equation: $g_{\mathrm{lum}} \approx 4|m|\cos\theta_{\mu,m}/|u|$. Hence, larger dissymmetry factor can be obtained through electrically forbidden (i.e., small $|\mu|$) and magnetically allowed (i.e., large $|m|$) transitions, such as the (n, π*) characteristic in carbonyls[19]. In view of factors above, the large luminescence dissymmetry factors achieved in the SGs could be largely attributed to the macroscopic self-assembly of Zn-L complexes, as well as the efficient (n, π*) state involved transition facilitated by the nitrogen and oxygen atoms.

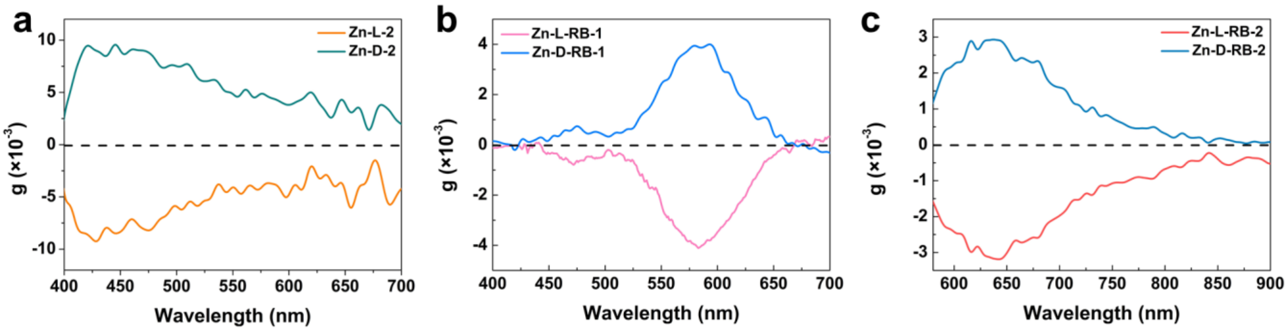

Supplementary Figure 27 | The CPL dissymmetry factors of the SGs. (a) CPL dissymmetry factors of Zn-L/D-2, (b) Zn-L/D-RB-1, and (c) Zn-L/D-RB-2 SGs.

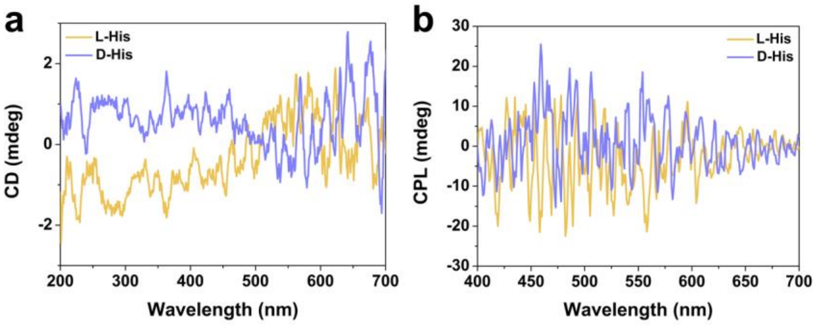

Supplementary Figure 28 | The CPL measurements of L/D-His powders. (a) CD and (b) CPL spectra of L/D-His powders excited by 320 nm.

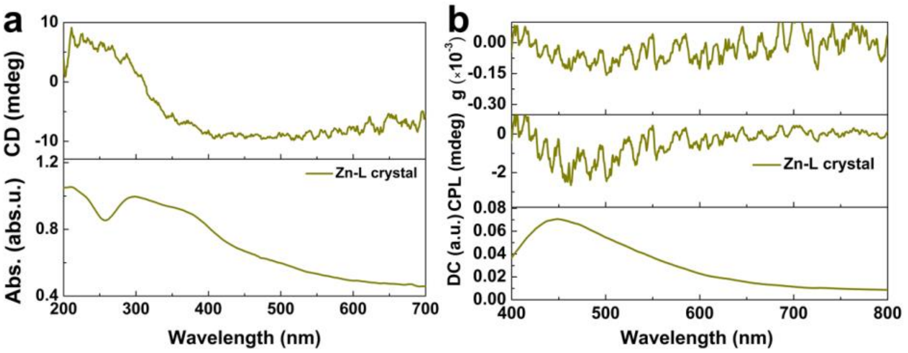

Supplementary Figure 29 | The CPL properties of Zn-L crystal. (a) CD and (b) CPL spectra and CPL dissymmetry factor of Zn-L crystal excited by 350 nm.

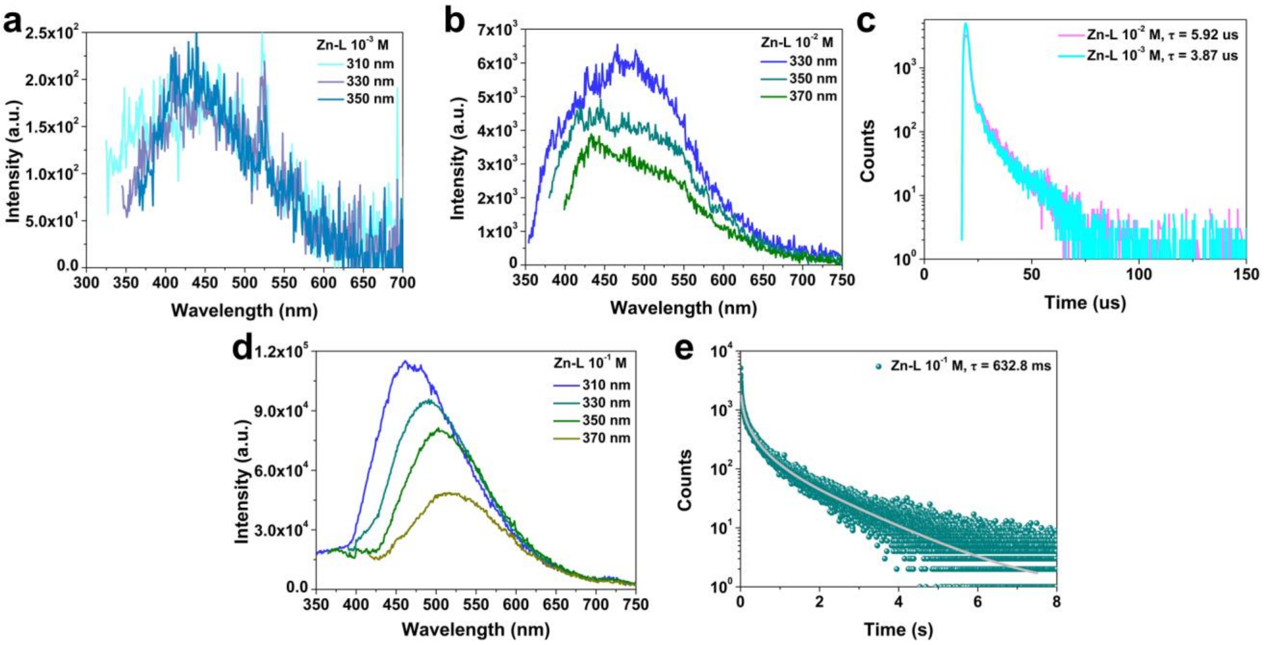

Supplementary Figure 30 | The luminescent properties of Zn-L complexes in aqueous solutions at 77 K.

(a, b) Delayed PL spectra of Zn-L complexes in aqueous solutions (10^{-3} and 10^{-2} M) excited by different wavelengths and (c) their phosphorescence decay curves at 478 and 430 nm excited by 350 nm at 77 K. (d) Delayed PL spectra of Zn-L complexes in aqueous solution (10^{-1} M) excited by different wavelengths, and (e) its phosphorescence decay curve at 505 nm excited by 350 nm at 77 K.

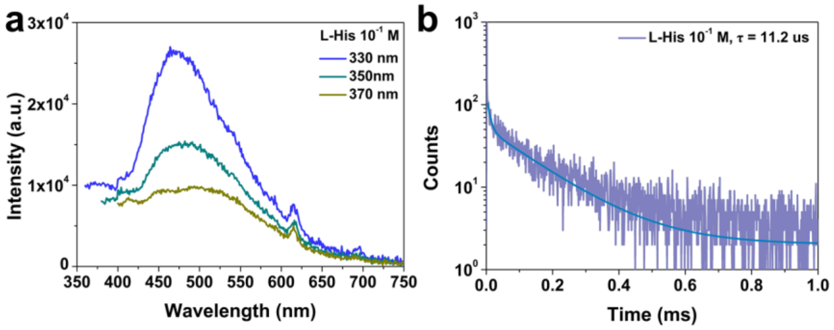

Supplementary Figure 31 | The PL behaviors of L-His aqueous solution at 77 K. (a) Delayed PL spectra of L-His in aqueous solution (10^{-1} M) excited by different wavelengths and (b) its phosphorescence decay curve at 482 nm excited by 350 nm at 77 K.

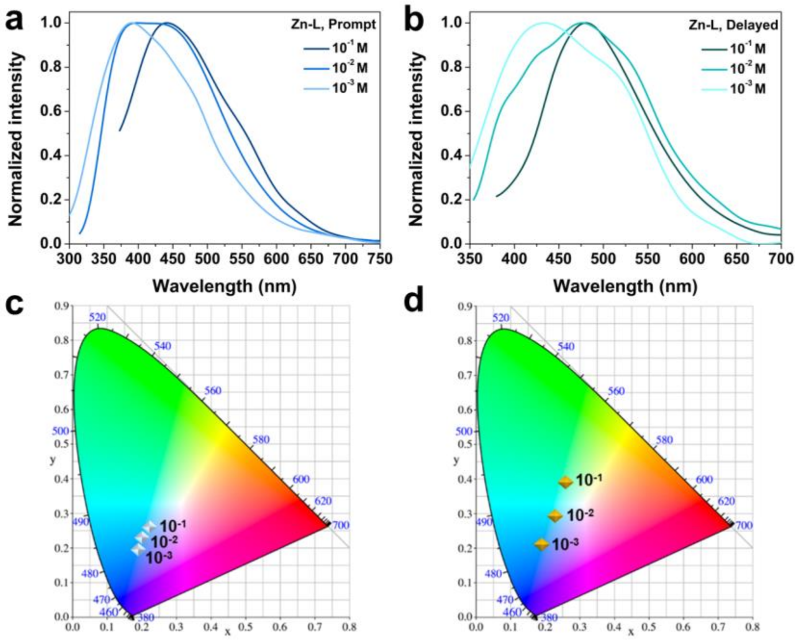

Supplementary Figure 32 | The PL behaviors of Zn-L complexes in aqueous solutions at 77 K. (a, b) Prompt and delayed PL spectra of Zn-L complexes in aqueous solutions (10^{-1}, 10^{-2} and 10^{-3} M) at 77 K excited by 280 and 350 nm, respectively, and (c, d) corresponding emission positions in the chromaticity coordinates diagrams.

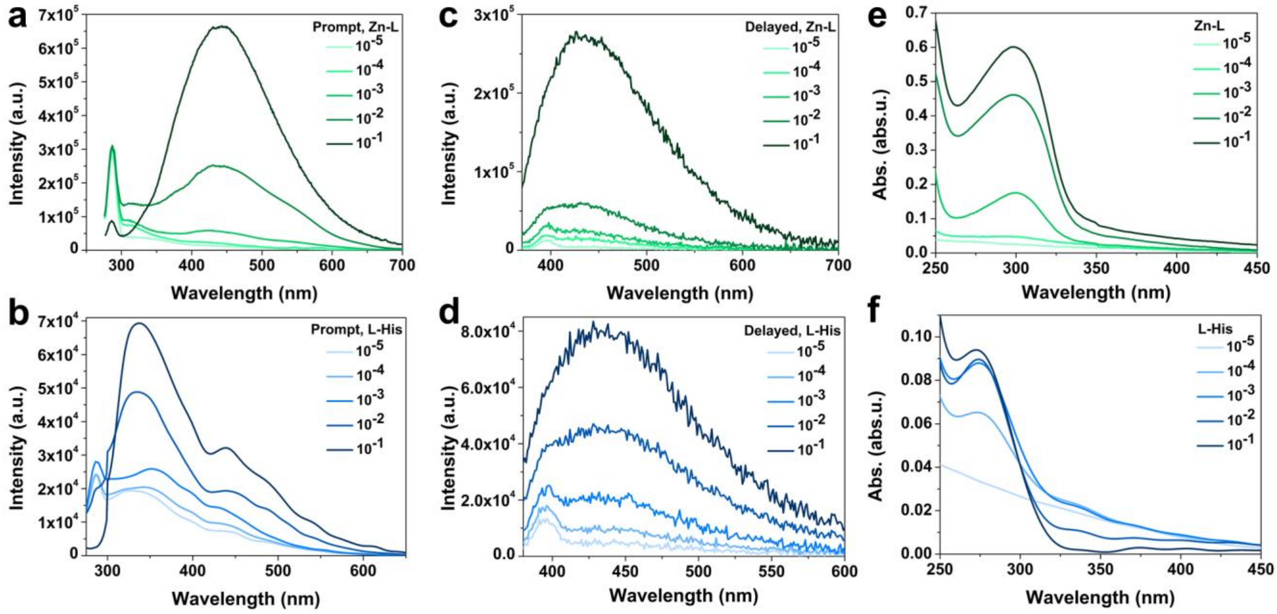

Supplementary Figure 33 | The photophysical properties of Zn-L complexes and L-His in aqueous solutions. (a, b) Prompt and (c, d) delayed PL spectra of Zn-L complexes and L-His molecules in aqueous solutions (10^{-1}–10^{-5} M) excited by 250 and 350 nm, respectively, and (e, f) their UV-Vis absorption spectra at room temperature.

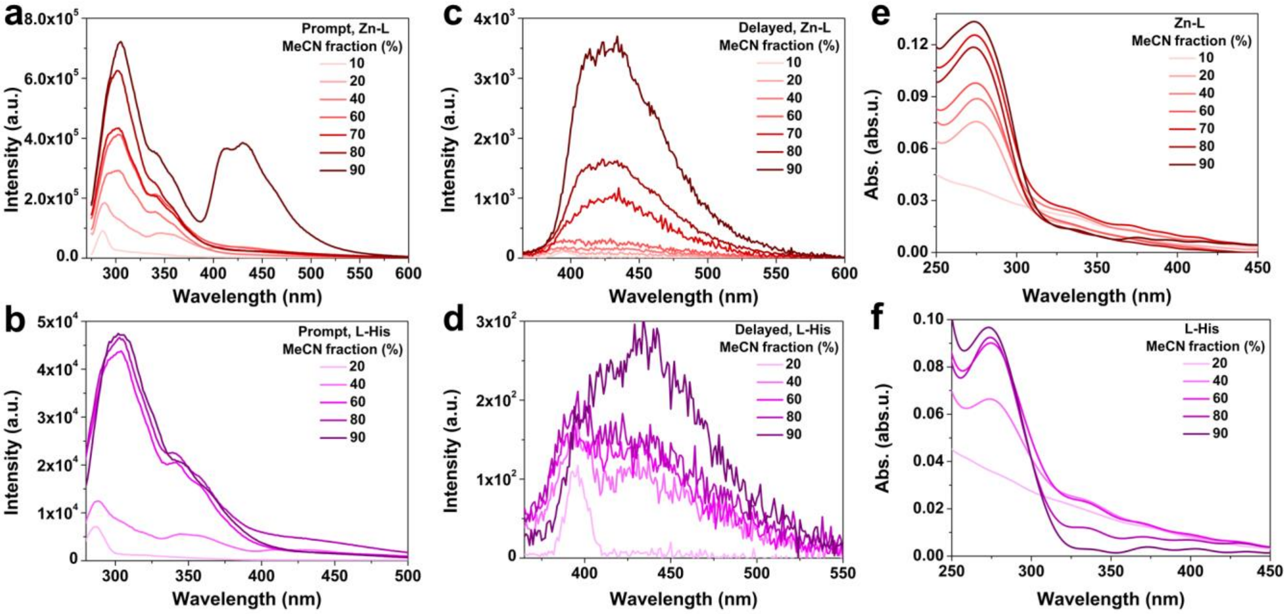

Supplementary Figure 34 | The photophysical properties of Zn-L complexes and L-His in H_2O/MeCN mixtures. (a, b) Prompt and (c, d) delayed PL spectra of Zn-L complexes and L-His molecules in H_2O/MeCN mixtures with different fractions of MeCN (10–90%) excited by 250 and 350 nm, respectively, and (e, f) their UV-Vis absorption spectra at room temperature.

Supplementary Note 18: The rationality statement of the PRET mechanism in the RB-doped SGs

Remarkably, a series of measurements have also yielded strong evidence for the proposed PRET mechanism. The strong RTP emission with high air stability (even after 5 months) of Zn-L-2 SG indicated a strong oscillator strength of the $T_1 \rightarrow S_0$ transition (Supplementary Figure 35c)[20]. It was speculated that the stable phosphorescence may benefit from strong SOC with the contribution of (n, π^*) states facilitated by nitrogen/oxygen heteroatoms. The strong SOC and consequent efficient RTP were crucially important, because the oscillator strength of phosphorescence in the donor served as a precondition for the dipole–dipole coupling between the excited donor and ground-state acceptor to promote the PRET process.

Time-resolved emission decay profiles of Zn-L-2 and Zn-L-RB-1/2 SGs showed the gradual decrease of the lifetime from 307.9, 176.3, to 141.9 ms upon increasing the RB-doping ratio from Zn-L-2, Zn-L-RB-1, to Zn-L-RB-2 SGs (Fig. 4), which hinted the non-existence of emission-reabsorption process and further supported the concept of PRET[21].

Furthermore, the prompt PL spectrum of RB powder exhibited the fluorescence emission peak at 688 nm (Supplementary Figure 35c), which nearly matched the delayed emission band at 678 nm of Zn-L-RB-2 SG. Meanwhile, the negligible emission in the delayed PL spectrum of RB may rule out the possibility of the emission at 688 nm from thermally activated delayed fluorescence. Collectively, these results strongly confirmed the rationality of the proposed PRET mechanism in the RB-doped SGs.

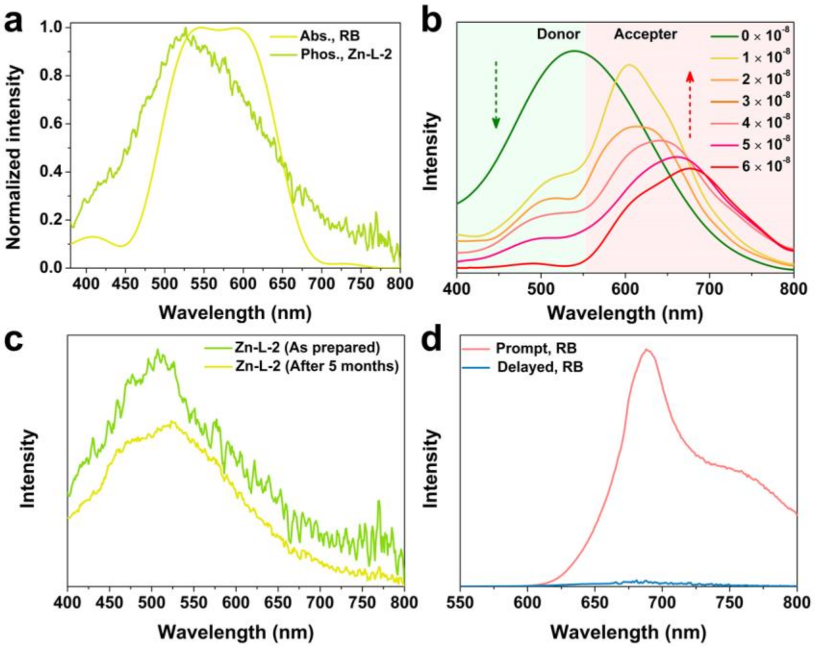

Supplementary Figure 35 | The experiments supporting the proposed PRET mechanism in the RB-doped SGs. (a) UV-Vis absorption spectrum of RB and phosphorescence spectrum of Zn-L-2 SG. (b) Delayed

PL spectra of RB doped SGs with different molar ratios of Zn-L complexes to RB (1:0–6 × 10^{-8}). (c) Delayed PL spectra of Zn-L-2 SG showing considerable phosphorescence intensity even after 5 months in air compared to the freshly prepared samples (λ_{ex} = 365 nm). (d) The prompt and delayed PL spectra of RB excited by 320 and 365 nm, respectively.

Supplementary Note 19: The results of frontier molecular orbital distributions for L-His monomer

Based on the simulation results of L-His monomer, the HOMOs and the LUMOs were separately localized on carboxylate group, and amino and imidazole groups, which could be ascribed to the typical LC mechanism.

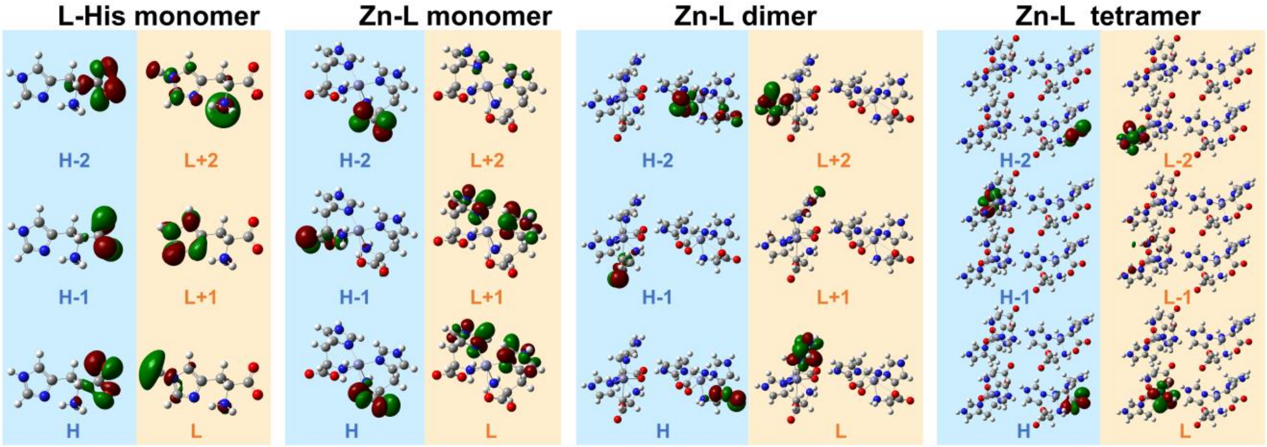

Supplementary Figure 36 | Frontier molecular orbital distributions for the selected L-His monomer extracted from L-His single crystal, and Zn-L monomer and aggregates extracted from Zn-L single crystal. H: HOMO, L: LUMO. The red and green regions of frontier molecular orbitals represent positive and negative phase values, respectively.

Supplementary Note 20: The results of ESP distribution maps for L-His monomer

In L-His monomer, the region around the carboxylate group represented the most negative potential region (blue), and the most positive potential site was largely around the hydrogen atoms linked with nitrogen atoms of imidazole and amino groups (red), implying the formation of the intermolecular hydrogen-bonding between the carboxylate and imidazole groups, as well as the carboxylate and amino groups. This analysis suggested that the hydrogen-bonding between L-His molecules basically remained between the L-His fractions in the coordination environment.

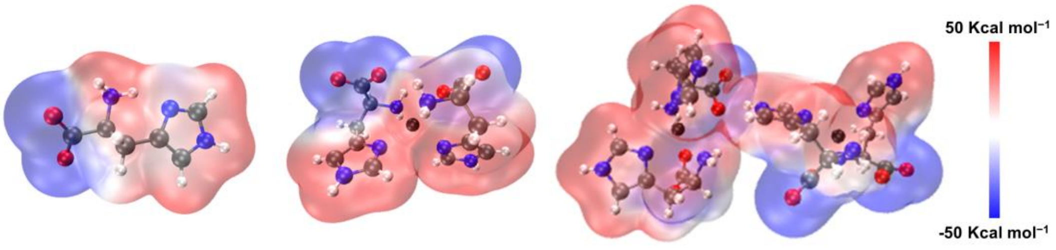

Supplementary Figure 37 | Calculated ESP distribution maps of selected L-His monomer in L-His single crystal and Zn-L monomer and dimer extracted from Zn-L single crystal. As indicated by the color bar, more positive (negative) ESP values correspond to reddish (bluish) colors.

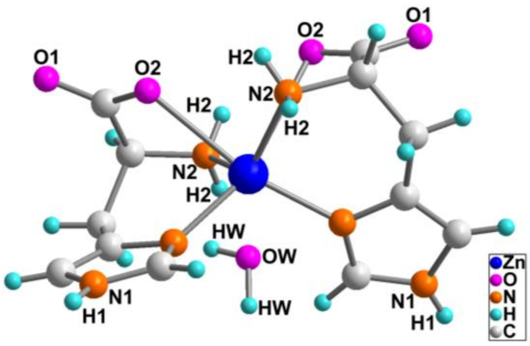

Supplementary Figure 38 | Modeling of Zn-L complex and water molecule.

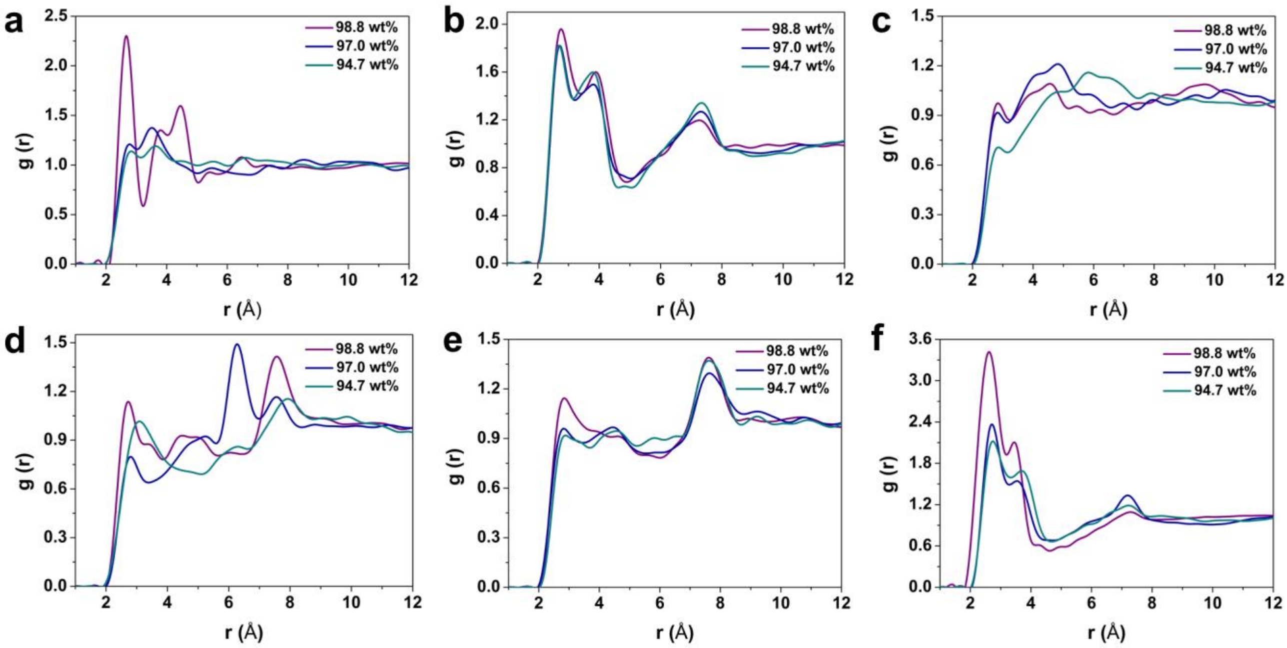

Supplementary Figure 39 | RDFs of hydrogen bonds between Zn-L complexes at different mass fractions of Zn-L complexes (98.8, 97.0 and 94.7 wt%. a: O1-H1, b: O1-H2, c: O2-H1, d: O2-H2, e: N1-H2, f: N2-H1.

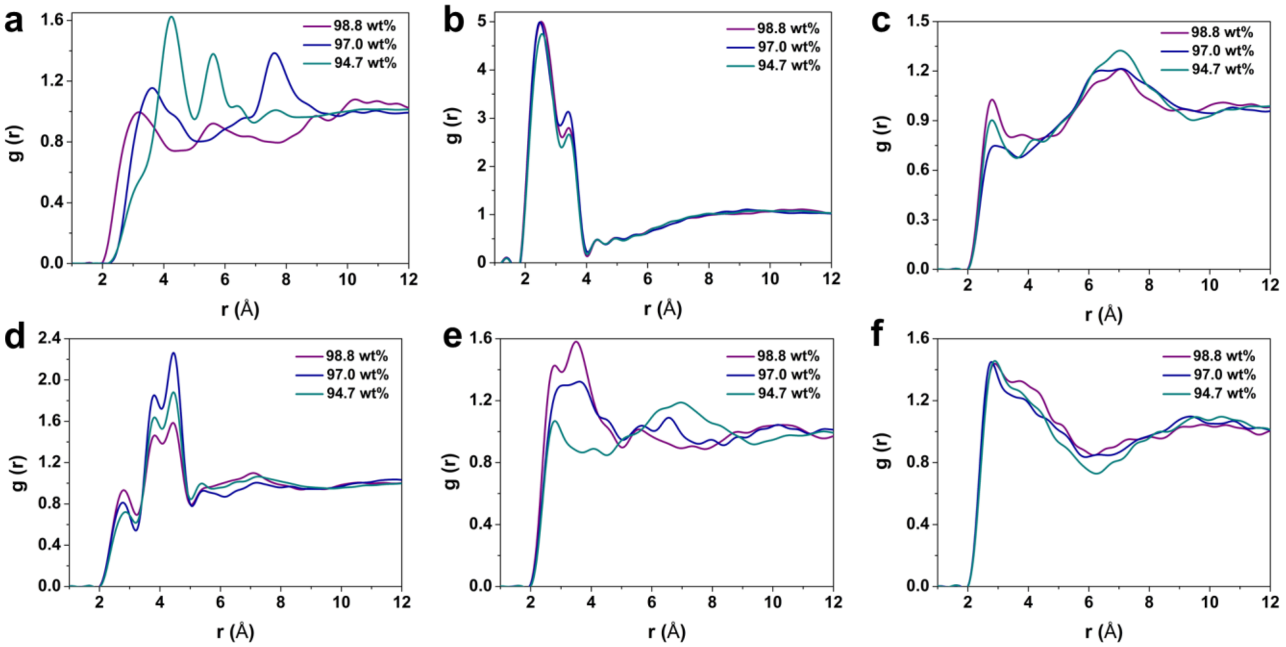

Supplementary Figure 40 | RDFs of hydrogen bonding between Zn-L complex and water molecule at different mass fractions of Zn-L complexes (98.8, 97.0 and 94.7 wt%). a: N1-HW, b: O1-HW, c: N2-HW, d: O2-HW, e: OW-H1, f: OW-H2.

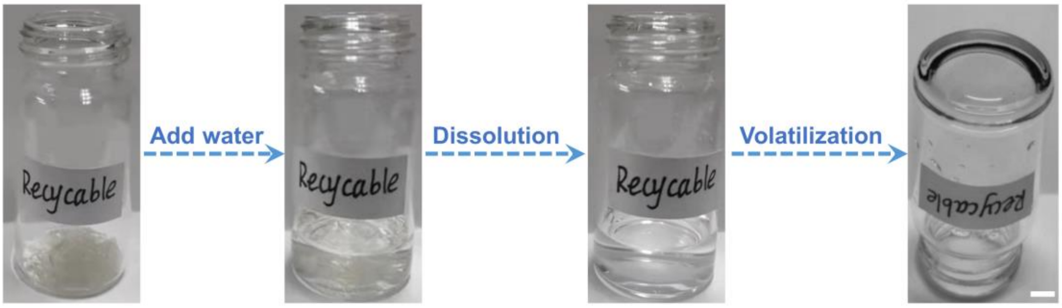

Supplementary Figure 41 | Photographs for the recycling process of the Zn-L SG fragments transformed into a large piece of glass. Scale bar: 0.4 cm.

Supplementary Table 1 | Crystal data for Zn-L crystal.

Sample*	Zn-L
**Molecular Formula**	$C_{12}H_{18}N_6O_6Zn$
**Molecular Weight**	407.69
**Crystal system**	Orthorhombic
**Space group**	$P2_12_12_1$
**a (Å)**	7.2850(4)
**b (Å)**	7.5558(5)
**c (Å)**	30.1842(11)
**α (deg)**	90.00
**β (deg)**	90.00
**γ (deg)**	90.00
**V (Å^3)**	1661.5(2)
**Density (g cm^{-3})**	1.630
**Z**	4
**S**	1.050
**R [I > 2σ(I)]**	$R_1 = 0.0887$
	$wR_2 = 0.2112$

*Crystallographic data have been deposited with the Cambridge Crystallographic Data Centre as supplementary publication no. CCDC: 2189962 for Zn-L crystal.

Supplementary Table 2 | Photoluminescence efficiencies (Φ_{PL}), phosphorescence time constants and the pre-exponential amplitudes of Zn-L-1/2/3/4, Zn-D-2 and Zn-L-RB-1/2 SGs as well as Zn-L crystal.

Sample	Φ_{PL} (%)	m	τ_i (ms)	A_i (%)	χ^2	<τ> (ms)
**Zn-L-1 SG**	18.32	3	8.32	12.45	1.002	356.7
			20.79	23.89		
			550.89	63.66		
**Zn-L-2 SG**	15.21	2	20.68	15.11	0.891	307.9
			359.02	84.89		

**Zn-L-3 SG**	11.77	2	9.95	18.79	0.903	224.2
			273.77	81.21		
**Zn-L-4 SG**	9.54	3	1.79	17.52	0.968	207.6
			28.76	24.84		
			347.23	57.64		
**Zn-D-2 SG**	16.02	2	148.82	24.37	0.904	315.5
			369.21	75.63		
**Zn-L-RB-1 SG**	20.31	2	34.50	23.69	0.895	176.3
			220.32	76.31		
**Zn-L-RB-2 SG**	21.59	3	3.07	6.89	1.119	141.9
			42.56	32.56		
			211.12	60.55		
**Zn-L crystal**	6.87	2	0.63	29.84	1.087	6.1
			8.45	70.16		

The m stands for the double- or three-exponential fitting of the PL decay curve, the τ_i represents the excited state lifetime, the A_i represents the ratio of τ_i, the value of χ^2 manifests the goodness of fitting, which is required to be below 1.300.

Supplementary Note 21: The results of TD-DFT calculations for L-His and Zn-L monomers

As we have known, the possible ISC process needs to follow at least two factors based on the energy gap law: on the one hand, S_1 and T_n should contain the same transition orbital component; on the other hand, the energy of plausible T_n should be in the range of $E_{S1} \pm 0.4$ eV[22]. According to the results of TD-DFT calculations of singlet and triplet excited state transition configurations of L-His and Zn-L monomers (Supplementary Table 3, 4), for Zn-L monomer, the transition configurations of the three excited triplet (T_1–T_3) were all contained in the transition configurations of the S_1, which greatly facilitated the transition of excitons from singlet to triplet states. However, for L-His monomer, although it exhibited six excited triplet states between $E_{S1} \pm 0.4$ eV, a very small proportion of the transition configurations of the T_1–T_6 were obtained in the transition configurations of the S_1. According to the theoretical and experimental results, it was deduced that there existed a higher probability of ISC progress in Zn-L monomer compared with that in L-His monomer, although the total number of plausible ISC channels in L-His monomer was more than that in Zn-L monomer.

Supplementary Table 3 | Singlet and triplet excited state transition configurations of L-His monomer revealed by TD-DFT calculations.

**Excited state**	***n*-th**	**Energy (eV)**	**Transition configuration (%)**
**S_n**	1	3.90	H→L (58.24), H→L+1 (16.11), H→L+2 (35.83)
**T_n**	1	3.84	H→L (54.85), H→L+1 (13.66), H→L+2 (40.92)
	2	4.18	H-3→L+1 (12.60), H-2→L (11.68), H→L (24.49), H→L+1 (43.39), H→L+2 (43.93)
	3	4.19	H-3→L+1 (18.11), H-2→L (23.91), H-2→L+2 (24.05), H→L (31.00), H→L+1 (42.34), H→L+2 (21.09)
	4	4.22	H-3→L+1 (16.57), H-2→L (34.20), H-2→L+2 (33.34), H-1→L (15.21), H-1→L+2 (14.45), H→L (19.96), H→L+1 (27.09), H→L+2 (26.09)
	5	4.26	H-3→L+1 (44.69), H-3→L+2 (21.01), H-3→L+6 (12.22), H-2→L (23.84), H-2→L+1 (26.15), H-2→L+2 (10.91), H-1→L+1 (13.04), H→L+1 (18.43), H→L+2 (13.16)
	6	4.26	H-2→L (14.17), H-2→L+2 (14.02), H-1→L (50.75), H-1→L+1 (14.36), H-1→L+2 (38.21)
	7	4.63	H-2→L (11.08), H-2→L+1 (14.26), H→L (40.69), H-1→L+1 (10.17), H-1→L+2 (51.79)
	8	4.64	H-2→L (22.14), H-2→L+1 (13.60), H-2→L+2 (25.40), H-1→L (10.59), H-1→L+1 (57.31)
	9	4.67	H-2→L (40.65), H-2→L+1 (15.82), H-2→L+2 (42.47), H-1→L (16.74), H-1→L+1 (27.02), H-1→L+2 (13.93)
	10	4.70	H-3→L+1 (23.42), H-3→L+2 (11.28), H-2→L+1 (57.82), H-2→L+2 (13.45), H-1→L+1 (16.84)

Supplementary Table 4 | Singlet and triplet excited state transition configurations of Zn-L monomer revealed by TD-DFT calculations.

**Excited state**	***n*-th**	**Energy (eV)**	**Transition configuration (%)**
**S_n**	1	2.99	H→L (50.08), H→L+1 (47.51), H→L+2 (14.99)
**T_n**	1	2.97	H→L (49.64), H→L+1 (48.41), H→L+2 (13.24)
	2	3.18	H→L (42.43), H→L+1 (50.32), H→L+2 (25.19)
	3	3.21	H→L (26.51), H→L+2 (63.77)
	4	3.47	H-1→L+1 (48.58), H-1→L+1 (47.15), H-1→L+2 (16.72)
	5	3.55	H→L+3 (65.05), H→L+4 (24.51), H→L+5 (10.93)
	6	3.62	H-2→L (47.00), H-2→L+1 (46.27), H-2→L+2 (12.09), H-1→L (11.91), H-1→L+1 (13.69)
	7	3.69	H-3→L (20.48), H-3→L+1 (20.18), H-2→L (13.52), H-2→L+1 (13.49), H-1→L (36.72), H-1→L+1 (44.71), H-1→L+2 (19.78)
	8	3.72	H-1→L (13.52), H-1→L+2 (34.54), H→L+3 (21.21), H→L+4 (53.74)
	9	3.74	H-3→L (12.87), H-3→L+1 (12.40), H-1→L (15.91), H-1→L+2 (53.50), H→L+3 (14.10), H→L+4 (34.05)
	10	3.75	H-3→L (42.35), H-3→L+1 (40.83), H-3→L+2 (11.49), H-1→L (25.37) H-1→L+1 (22.11), H-1→L+2 (10.27)

Supplementary Table 5 | Singlet and triplet excited state transition configurations of Zn-L dimer revealed by TD-DFT calculations.

**Excited state**	***n*-th**	**Energy (eV)**	**Transition configuration (%)**
S_n	1	2.76	H-1→L (70.55)
T_n	1	2.58	H→L (70.68)
	2	2.95	H→L+1(70.54)
	3	2.95	H→L+1 (69.51), H→L+3 (11.66)
	4	2.97	H-2→L+2 (70.26)
	5	3.02	H→L+4 (70.42)
	6	3.05	H-1→L+2 (70.69)
	7	3.08	H-3→L (21.47), H-2→L+1 (66.77)
	8	3.15	H-1→L+1 (67.84), H-1→L+3 (17.15)
	9	3.19	H→L+1 (12.41), H→L+3 (65.20), H→L+6 (24.01)
	10	3.26	H-3→L+1 (67.18), H-2→L (21.90)
	11	3.32	H→L+3 (24.18), H→L+6 (65.01)
	12	3.35	H-4→L (69.92)
	13	3.41	H-1→L+1 (16.61), H-1→L+3 (67.85)
	14	3.45	H-5→L (70.54)
	15	3.45	H-3→L+1 (20.48), H-2→L+1 (63.91), H-2→L+3 (17.41)
	16	3.49	H→L+5 (68.40), H→L+7 (14.38)
	17	3.53	H-6→L (70.43)
	18	3.59	H-3→L+2 (20.13), H-2→L+2 (67.32)
	19	3.61	H-8→L+2 (16.65), H-7→L (68.64)

	20	3.62	H-5→L+2 (12.03), H-1→L+5 (67.43)

Supplementary references

1. Debenedetti, P.G. and Stillinger, F.H. Supercooled liquids and the glass transition. *Nature* **410,** 259−267 (2001).
2. Rodrigues, A.M., Cassar, D.R., Fokin, V.M. and Zanotto, E.D. Crystal growth and viscous flow in barium disilicate glass. *J. Non-Cryst. Solids* **479,** 55−61 (2018).
3. Sun, Z.P., Guo, Y.B. and Shim, V.P.W. Characterisation and modeling of additively-manufactured polymeric hybrid lattice structures for energy absorption. *Int. J. Mech. Sci*. **191,** 106101 (2021).
4. Sun, Y. et al. The variation in elastic modulus throughout the compression of foam materials. *Acta Mater*. **110,** 161–174 (2016).
5. Feng, G., Li, S., Xiao, L. and Song, W. Energy absorption performance of honeycombs with curved cell walls under quasi-static compression. *Int. J. Mech. Sci.* **210,** 106746 (2021).
6. Liu, L. and Dai, J. C. [Zn(tp)(RdmB)(H_2O)] and [Cd(tp)(RdmB)]: Two Unusual One-Dimensional Rhodamine B Coordination Polymeric Ribbons as Luminescent Sensors for Small Molecules and Metal Cations. *Cryst. Growth Des*. **18,** 4460−4469 (2018).
7. Qiao, A. et al. A metal-organic framework with ultrahigh glass-forming ability. *Sci. Adv*. **4,** eaao6827 (2018).
8. Hidaka, J., Yamada, S. and Shimura, Y. Preparation of the Isomers of Bis(L-Methioninato)cobalt (III) Bromide. *Chem. Lett.* **3,** 1487–1490 (1974).
9. Watabe, M., Yano, H. and Yoshikawa, S. Preparation and Isomerization of Isomers of the L- or D-Aspartato(L-histidinato)cobalt(III) Complex. *Bull. Chem. Soc. Jpn*. **52,** 61–64 (1979).
10. Stevenson, B. C. et al. An investigation of inter-ligand coordination and flexibility: IRMPD spectroscopic and theoretical evaluation of calcium and nickel histidine dimers. *J. Mol. Spectrosc.* **381,** 111532 (2021).
11. Stevenson, B. C. et al. IRMPD Spectroscopic and Theoretical Structural Investigations of Zinc and Cadmium Dications Bound to Histidine Dimers. *J. Phys. Chem. A* **124,** 10266–10276 (2020).
12. Hofstetter, T. E., Howder, C., Berden, G., Oomens, J. and Armentrout, P. B. Structural Elucidation of Biological and Toxicological Complexes: Investigation of Monomeric and Dimeric Complexes of

Histidine with Multiply Charged Transition Metal (Zn and Cd) Cations using IR Action Spectroscopy. *J. Phys. Chem. B* **115,** 12648–12661 (2011).

13. De Silva, A., Felix, N. M. and Ober, C. K. Molecular Glass Resists as High-Resolution Patterning Materials. *Adv. Mater.* **20,** 3355–3361 (2008).
14. Shirota, Y. Organic materials for electronic and optoelectronic devices. *J. Mater. Chem.* **10,** 1–25 (2000).
15. Sasaki, Y., Yamamoto, T. and Mori, H. Mechanically robust, ion-conductive, self-healing glassy hybrid materials via tailored Zn/imidazole interaction. *Mater. Today Chem.* **22,** 100611 (2021).
16. Niazi, A., Ghalie, M., Yazdanipour, A. and Ghasemi, J. Spectrophotometric determination of acidity constants of Alizarine Red S in water, water-Brij-35 and water-SDS micellar media solutions. *Spectrochim. Acta A* **64,** 660–664 (2006).
17. Abedin, F., Ye, Q. and Spencer, P. Hydrophilic dyes as photosensitizers for photopolymerization of dental Adhesives. *J. Dent.* **99,** 103405 (2020).
18. Takaishi, K., Murakami, S., Yoshinami, F. and Ema, T. Binaphthyl-Bridged Pyrenophanes: Intense Circularly Polarized Luminescence Based on a D_2 Symmetry Strategy. *Angew. Chem. Int. Ed.* **61,** e202204609 (2022).
19. Mori, T. Chiroptical Properties of Symmetric Double, Triple, and Multiple Helicenes. *Chem. Rev.* **121,** 2373−2412 (2021).
20. Kuila, S. and George, S. J. Phosphorescence Energy Transfer: Ambient Afterglow Fluorescence from Water-Processable and Purely Organic Dyes via Delayed Sensitization. *Angew. Chem. Int. Ed.* **59,** 9393–9397 (2020).
21. Zhao, Y. et al. Visible Light Activated Organic Room Temperature Phosphorescence Based on Triplet-to-Singlet Förster Resonance Energy Transfer. *Adv. Optical Mater.* **10,** 2102701 (2022).
22. Fang, M. et al. Unexpected room-temperature phosphorescence from a non-aromatic, low molecular weight, pure organic molecule through the intermolecular hydrogen bond. *Mater. Chem. Front.* **2,** 2124–2129 (2018).
