# Supplementary material for: Supramolecular glasses with color-tunable circularly polarized afterglow through evaporation-induced self-assembly of chiral metal–organic complexes
Source: Nat Commun. 2023 Mar 24;14:1654. doi: 10.1038/s41467-023-37331-0 (PMC10039082; doi:10.1038/s41467-023-37331-0)
Supplement: Supplementary file 2 — Description of Additional Supplementary Files [file 41467_2023_37331_MOESM2_ESM.pdf]

Supplementary Data 1

The crystallographic data of Zn-L crystal (CCDC number: 2189962).

Supplementary Movie 1

Luminescence behaviors of the round sheet of Zn-L-2 SG under the irradiations of a UV lamp at 365 and 395 nm.

Supplementary Movie 2

Luminescence behaviors of spherical Zn-L-2 SG under the irradiations of a UV lamp at 365 and 395 nm.

Supplementary Movie 3

Luminescence behavior of spherical Zn-L-RB-1 SG under the irradiation of a UV lamp at 365 nm.

Supplementary Movie 4

Luminescence behavior of spherical Zn-L-RB-2 SG under the irradiation of a UV lamp at 365 nm.
